# Assessing the relative contribution of CYP3A-and P-gp-mediated pathways to the overall disposition and drug-drug interaction of dabigatran etexilate using a comprehensive mechanistic physiological-based pharmacokinetic model

**DOI:** 10.3389/fphar.2024.1356273

**Published:** 2024-03-07

**Authors:** Udomsak Udomnilobol, Wilasinee Dunkoksung, Watchara Sakares, Suree Jianmongkol, Thomayant Prueksaritanont

**Affiliations:** ^1^ Chulalongkorn University Drug Discovery and Drug Development Research Center (Chula4DR), Chulalongkorn University, Bangkok, Thailand; ^2^ Department of Pharmacology and Physiology, Faculty of Pharmaceutical Sciences, Chulalongkorn University, Bangkok, Thailand

**Keywords:** CYP3A, dabigatran, drug-drug interaction, microdose, nonlinear pharmacokinetics, P-glycoprotein, physiologically based pharmacokinetics

## Abstract

Dabigatran etexilate (DABE) is a clinical probe substrate for studying drug-drug interaction (DDI) through an intestinal P-glycoprotein (P-gp). A recent *in vitro* study, however, has suggested a potentially significant involvement of CYP3A-mediated oxidative metabolism of DABE and its intermediate monoester BIBR0951 in DDI following microdose administration of DABE. In this study, the relative significance of CYP3A- and P-gp-mediated pathways to the overall disposition of DABE has been explored using mechanistic physiologically based pharmacokinetic (PBPK) modeling approach. The developed PBPK model linked DABE with its 2 intermediate (BIBR0951 and BIBR1087) and active (dabigatran, DAB) metabolites, and with all relevant drug-specific properties known to date included. The model was successfully qualified against several datasets of DABE single/multiple dose pharmacokinetics and DDIs with CYP3A/P-gp inhibitors. Simulations using the qualified model supported that the intestinal CYP3A-mediated oxidation of BIBR0951, and not the gut P-gp-mediated efflux of DABE, was a key contributing factor to an observed difference in the DDI magnitude following the micro-versus therapeutic doses of DABE with clarithromycin. Both the saturable CYP3A-mediated metabolism of BIBR0951 and the solubility-limited DABE absorption contributed to the relatively modest nonlinearity in DAB exposure observed with increasing doses of DABE. Furthermore, the results suggested a limited role of the gut P-gp, but an appreciable, albeit small, contribution of gut CYP3A in mediating the DDIs following the therapeutic dose of DABE with dual CYP3A/P-gp inhibitors. Thus, a possibility exists for a varying extent of CYP3A involvement when using DABE as a clinical probe in the DDI assessment, across DABE dose levels.

## Introduction

Dabigatran etexilate (DABE) is a clinical probe substrate used to phenotype intestinal P-glycoprotein (P-gp) function either in drug-drug interaction (DDI) or pharmacokinetic (PK) studies in special populations (EMA, 2012; Chu et al., 2018; FDA, 2020). It is a double ester prodrug of a pharmacologically active moiety, dabigatran (DAB) (FDA, 2010b). After absorption, DABE is known to be rapidly hydrolyzed by a carboxylesterase (CES)2 enzyme in the intestine to form two parallel intermediate metabolites, dabigatran ethylester (BIBR0951) and desethyl dabigatran Etexilate (BIBR1087), which are further converted to DAB by hepatic CES1/2 enzymes (Laizure et al., 2014; Laizure et al., 2022). In contrast to the parent DABE, these two metabolites (BIBR0951 and BIBR1087) and DAB are not substrates of P-gp (FDA, 2010b). We have recently demonstrated that in human intestinal microsomes (HIM), DABE underwent NADPH-dependent oxidation in parallel to CES-mediated hydrolysis (Udomnilobol et al., 2023). Furthermore, NADPH-dependent metabolism of its intermediate monoester BIBR0951 was also observed in both HIM and human liver microsomes (HLM) (Udomnilobol et al., 2023). The oxidative metabolism of DABE and BIBR0951, which was almost exclusively via CYP3A, was saturable, with K_m_ values (1–3 µM) significantly below the expected concentrations after administration of a therapeutic dose of DABE. The findings provided a likely explanation for the apparent overestimation of DDI magnitude observed with the CYP3A/P-gp inhibitors following a microdose versus therapeutic dose of DABE (Prueksaritanont et al., 2017).

To date, several semi-mechanistic physiologically based pharmacokinetic (PBPK) models of DABE and DAB have been developed for various PK applications, including DDI prediction, formulation development, or PK prediction in renal impairment (Zhao and Hu, 2014; Doki et al., 2019; Moj et al., 2019; Yamazaki et al., 2019; Wang et al., 2020; Farhan et al., 2021; Lang et al., 2021). However, none of the PBPK models mentioned above have included two intermediate metabolites (BIBR0951 and BIBR1087), due probably to limited knowledge on their disposition pathways. Furthermore, the CYP3A-mediated pre-systemic metabolism of both DABE and BIBR0951 (Udomnilobol et al., 2023) has never been considered in any published models; thus, the relative contribution of the intestinal P-gp- versus CYP3A-mediated pathways to the overall disposition of the microdose DABE remains to be determined.

Therefore, to help delineate the relative significance of these pathways to the overall disposition of DABE and thus the DDIs following both the microdose and therapeutic dose of DABE, we aimed to develop in this study a comprehensive mechanistic PBPK model of DABE linking its two intermediate metabolites to the eventual product of interest, DAB. All relevant drug-specific properties known to date, including biopharmaceutic properties of DABE, gut P-gp mediated DABE efflux, and CYP3A4/5-mediated oxidation of both DABE and BIBR0951, were incorporated into the model. After development, the model was qualified and subsequently applied to obtain mechanistic insights into the relative contribution of the P-gp- versus CYP3A involvement in the overall disposition and DDIs across dose levels of DABE.

## Materials and methods

### *In vitro* studies

Several values related to physicochemical and ADME properties of BIBR0951 and BIBR1087, including plasma stability, plasma protein binding, blood-to-plasma partitioning, enzyme kinetic, and permeability/transport) were determined by *in vitro* experiments, as described in the section of Supplementary Methods.

### PBPK software

Simcyp population based ADME simulator software version 20 (Certara, Sheffield, United Kingdom) was used for the PBPK analysis in this study. All simulations were performed using a virtual “Sim-Healthy Volunteer” population aged 20–50 years for 100 subjects (10 subjects per trial, 10 trials per run). Other parameters for the virtual trial design were set to mimic the clinical designs of observed data as much as possible.

### Clinical data for modeling and simulation

Supplementary Table S1 summarizes the observed datasets for model development and qualification. The clinical PK profiles and parameters of free DAB (unconjugated DAB) were gathered and included in this study. All observed plasma concentration-time profiles data were digitized from the published literature using DigitizeIt software version 2.5.9 (I. Bormann, Braunschweig, Germany). The full plasma concentration-time profiles and associated PK parameters of DABE and two intermediate metabolites (BIBR0951 and BIBR1087) were not available in the literature.

### Model development and qualification

**FIGURE 1:**
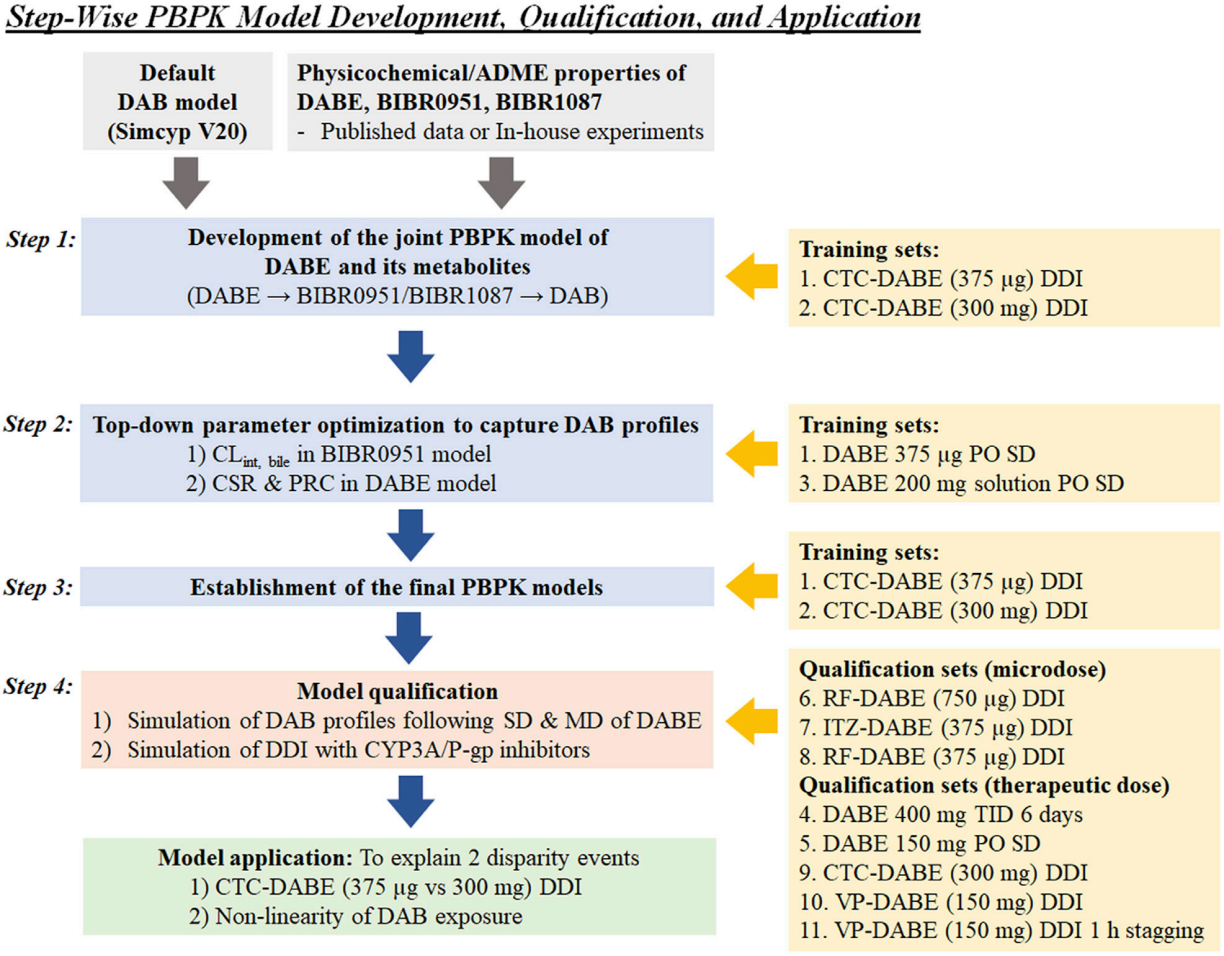
Stepwise processes of PBPK model development, qualification, and application used in this study. CSR, critical supersaturation ratio; CTC, clarithromycin; DAB, dabigatran; DABE, dabigatran etexilate; ITZ, itraconazole; PRC, precipitation rate constant; RF, rifampicin; VP, verapamil.

Figure 1 illustrates the stepwise processes for PBPK model development, qualification, and application.

Step 1 Development of the jointed PBPK model of DABE with its metabolites

**FIGURE 2:**
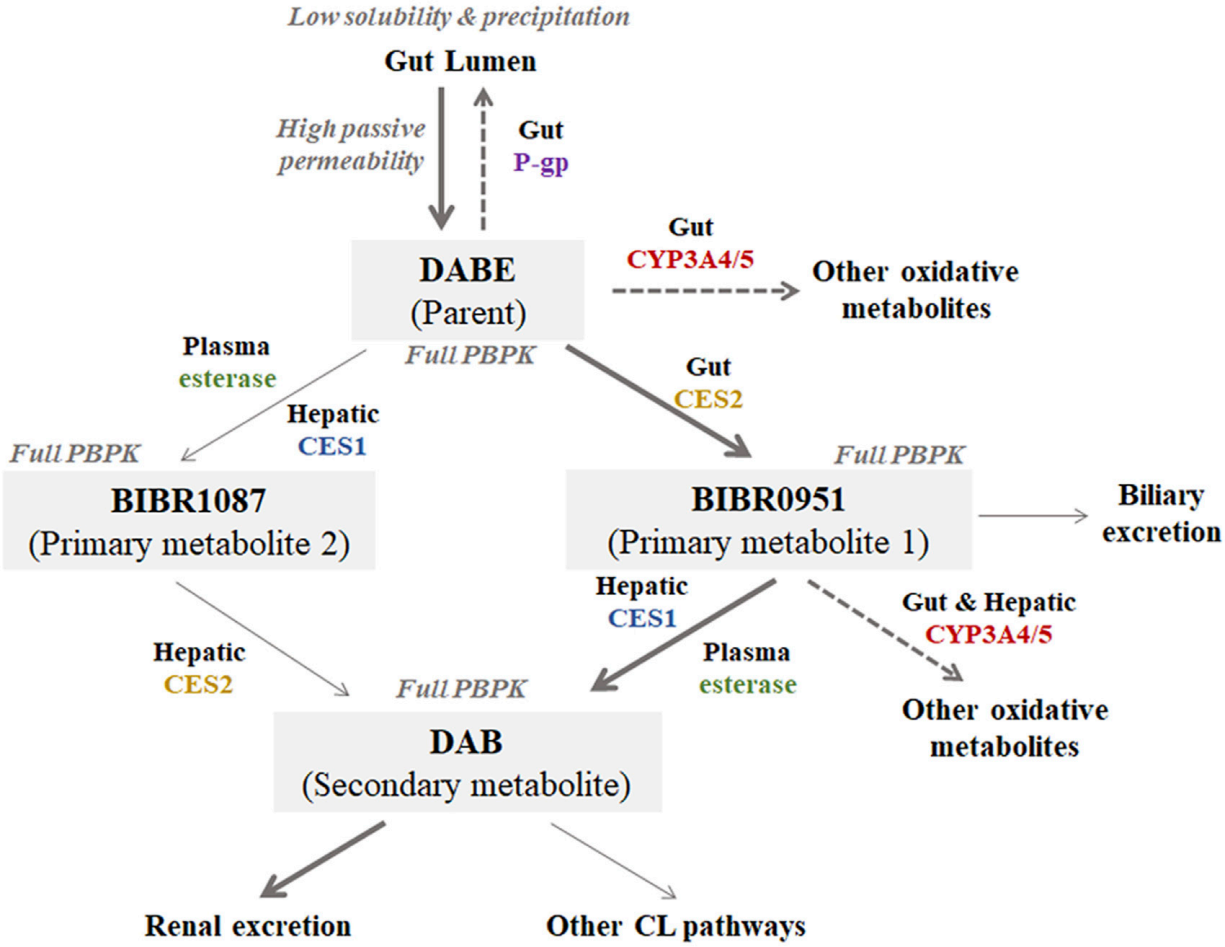
Structure of the jointed PBPK models of DABE with its metabolites (BIBR1087, BIBR0951, and DAB) proposed in this study. Bolded and light arrows represent the major and minor pathways, respectively. Dashed arrows represent the saturable pathways potentially contributing to disparity in DDI magnitudes between microdose and therapeutic dose DABE administration.

A mechanistically jointed PBPK model of DABE composed of four PBPK sub-models of DABE and its metabolites (BIBR0951, BIBR1087, and DAB) connected in a sequential fashion, similar to what occurs in humans (Figure 2). The DAB model was adopted from a default model (SV-Dabigatran) in the software without any modification. The PBPK models of DABE, BIBR0951, and BIBR1087 were newly developed via a middle-out approach, based on both *in vitro* and observed clinical data.

For the BIBR0951 model, the intestinal disposition of BIBR0951 was described using an Advanced Dissolution, Absorption, and Metabolism (ADAM) model (Ezuruik et al., 2021). An effective permeability coefficient in humans (P_eff,man_), which relates to the transport rate/permeability across the enterocytes, of BIBR0951 was predicted based on the polar surface area (PSA) and hydrogen bond donor (HBD) at 137 and 3, respectively, (PubChem, 2023), and used in the ADAM model to predict drug absorption. Based on the observed profiles, a basolateral (BL) global scalar governing the passing of the gut formed BIBR0951 into the hepatic portal vein was optimized to 0.1 unit, (Delavenne et al., 2013; Prueksaritanont et al., 2017). A whole-body distribution PBPK model was applied for prediction of steady-state volume of distribution (V_d,ss_) (Rodgers and Rowland, 2007). The metabolic conversion of BIBR0951 to DAB was governed by the kinetics of two hydrolytic pathways: 1) CES1-mediated hydrolysis using the kinetic parameters of BIBR0951 hydrolysis in HLM (Udomnilobol et al., 2023); and 2) plasma esterase-mediated hydrolysis using an *in vitro* half-life of BIBR0951 disappearance in the human plasma. According to previous *in vitro* findings in which gut and hepatic CYP3A significantly metabolized BIBR0951 to several oxidative metabolites (Udomnilobol et al., 2023), the enzyme kinetic parameters for CYP3A4/5-mediated BIBR0951 metabolism were incorporated into the model, as the parallel pathways competing with the DAB formation pathway. Intersystem extrapolation factors (ISEF) for CYP3A4/5 enzymes were calculated based on the *in vitro* metabolism data in HLM and recombinant enzymes (Udomnilobol et al., 2023), and used in the extrapolation of *in vitro* to *in vivo* CL parameters (Supplementary Methods).

For the BIBR1087 model, the V_d,ss_ value was predicted from the whole-body distribution model (Rodgers and Rowland, 2007). The hydrolysis of BIBR1087 to form DAB was mediated by the kinetics of CES1 and CES2 enzymes.

For the DABE model, all physicochemical properties were obtained from the published literature (FDA, 2010b; Doki et al., 2019). An intestinal absorption of DABE was described by the ADAM model using an *in vitro* passive apparent permeability coefficient in Caco-2 cells (P_app,caco-2_) as an input parameter (Ishiguro et al., 2014). Distribution of DABE was described by the whole-body distribution model (Rodgers and Rowland, 2007). Enzyme kinetic parameters of CES1- and CES2-mediated DABE hydrolysis were obtained from the previous literature (Udomnilobol et al., 2023), and incorporated into the model for driving the formation of 2 primary metabolites (BIBR0951 and BIBR1087). In addition, the *in vitro* half-life of plasma esterase-mediated hydrolysis was also considered for the conversion of DABE to BIBR1087. As shown previously that intestinal CYP3A significantly metabolized DABE to several oxidative products (Udomnilobol et al., 2023), the kinetic parameters of CYP3A4/5-mediated DABE metabolism was added into the model as a parallel pathway competing with CES1/2 hydrolysis. The ISEF values for CYP3A4/5 enzymes were calculated based on the *in vitro* metabolism data in HIM and recombinant systems (Udomnilobol et al., 2023). To model transporter-mediated DABE efflux, the P-gp K_m_ was fixed at 2.6 µM according to previously reported values (Yamazaki et al., 2019; Lang et al., 2021). The maximum transport rate (J_max_) of P-gp was then top-down estimated from the observed data (Delavenne et al., 2013; Prueksaritanont et al., 2017).

Step 2 Top-down parameter optimization to capture DAB profiles

To capture the plasma DAB profiles following microdose DABE administration, a global sensitivity analysis (GSA) using Morris’s method was utilized for screening of additional input parameters highly influencing the plasma profiles of DAB. The intrinsic clearance for biliary excretion of BIBR0951 was top-down estimated by fitting DAB PK profile following microdose administration of DABE (Prueksaritanont et al., 2017), using Simcyp-plugin parameter estimation (PE) function to obtain the best model-fitted intrinsic clearance value.

To capture the plasma DAB profiles following DABE administration at therapeutic dose, the input parameters related to solubility, precipitation, and formulation were added into the DABE model. Either solution with precipitation or solid immediate release (IR) dosage form was selected as the formulations of DABE, depending on the clinical study design (Supplementary Table S1). A diffusion layer model (DLM) was used for modeling dissolution and precipitation of DABE in the gut lumen (Farhan et al., 2021). An aqueous phase solubility of DABE at pH 7.4 was obtained from a published value of 0.003 mg/mL (FDA, 2010a). Parameters controlling the precipitation of DABE, including the critical supersaturation ratio (CSR) and precipitation rate constant (PRC), were top-down estimated from the observed plasma DAB profile following oral administration of 200 mg DABE solution (Blech et al., 2008).

Step 3 Establishment of the final PBPK models

The final PBPK models of DABE and its metabolites were re-evaluated using the DABE-CTC interaction dataset following microdose and therapeutic dose of DABE (Delavenne et al., 2013; Prueksaritanont et al., 2017). Supplementary Tables S2–S4 list the final input parameters for all drug models.

Step 4 PBPK model qualification

The developed models were qualified by simulating several clinical PK scenarios including the single-dose (SD) PK, multiple dose (MD) PK, and DDI with CYP3A/P-gp inhibitors (CTC; itraconazole, ITZ; rifampin, RF and verapamil, VP). The simulated results were then compared to the observed qualification datasets (Supplementary Table S1). The satisfactory qualification was justified based on the pre-specified criteria, described in the “*Model evaluation*” section.

For inhibitor PBPK models, the ITZ and its metabolite hydroxy-itraconazole (OH-ITZ) models were modified from Chen et al. (2019) by addition of inhibitory constants (K_i_) of ITZ and OH-ITZ for intestinal P-gp of 2 and 5 μM, respectively (Lang et al., 2021). For other perpetrators, the default models in the software were adopted without modification. The final input parameters for all inhibitor PBPK models are listed in Supplementary Table S5. Based on the final parameters and the dosing regimens (SD or MD), all the inhibitors employed were considered dual inhibitors of CYP3A (reversible and/or mechanism-based) and P-gp, but not inhibitors of CES1/2.

### Parameter estimation and sensitivity analysis

A top-down estimation was carried out via a default Simcyp-plugin parameter estimation (PE) module. Weighted least square and Nelder-Mead methods were chosen as the objective function and minimization method, respectively.

A sensitivity analysis of multiple input parameters was performed by a global sensitivity analysis (GSA) using Morris’s method. The numbers of levels, trajectories, and replications were set as default values in the software. The higher µ* value indicates greater importance of that parameter to overall simulation outputs.

### Model evaluation

Evaluation of PBPK models at all steps was justified based on the following three pre-specified criteria: 1) visual predictive check (VPC), 2) acceptance criteria for PK parameters, and 3) acceptance criteria for DDI prediction. For VPC, all observed plasma concentrations of DAB at various time points should be within the 5%–95% confidence interval of the simulated results. For acceptance criteria of plasma PK parameters (C_max_ and AUC_0-inf_), the alternative success criteria at a 99.998% confidence interval for PK parameters were calculated from the observed mean and percentage coefficient of variation (% CV) (Abduljalil et al., 2014). The prediction of PK parameters was considered successful when the observed PK parameters were within the lower and upper boundaries. For the datasets without standard deviation (s.d.) or % CV, the simulated PK parameters should be within twofolds of the observed data. For DDI prediction, the satisfactory prediction for DDI magnitudes was evaluated based on Guest’s DDI prediction criteria (Guest et al., 2011). The predicted C_max_ and AUC_0-inf_ ratios should be within the lower and upper limits of Guest’s acceptance range. The precision and bias for overall prediction were evaluated by calculating the geometric mean fold error (GMFE) using a formula: GMFE = $10^{\;\frac{\sum \left|\log\frac{s\text{imulated}}{\text{observed}}\right|}{\text{number}\;\text{of}\;\text{scenarios}}}$ . A model with good precision and no bias should have a GMFE within the range of 0.80–1.25.

## Results

### *In vitro* studies for supporting model development

To determine the input parameters for hydrolysis of DABE and its intermediate metabolites (BIBR0951 and BIBR1087) in human plasma, the compounds were separately incubated in the plasma for up to 2 h. Following incubation at 1 and 100 µM of DABE and BIBR0951, there was no obvious difference in parent disappearance or formation of hydrolytic products in the plasma between the 2 drug concentrations tested (Supplementary Figures S1A, B). The mean t_1/2_ values of DABE and BIBR0951 in plasma were at 364 and 55 min, respectively. These results showed that DABE and BIBR0951 were hydrolyzed in human plasma in a concentration-independent manner. In contrast, BIBR1087 was stable up to 2 h in human plasma (Supplementary Figure S1C).

For modeling BIBR1087 elimination, the kinetic parameters of BIBR1087 hydrolysis were determined based on the metabolite formation in HIM and HLM. The CES-mediated BIBR1087 hydrolysis was described by the Michaelis-Menten kinetics, with the V_max_ and K_m_ values shown in Supplementary Figure S2.

As shown in Supplementary Table S3, the fractions unbound in plasma (f_u,p_) of BIBR0951 and BIBR1087 were at 0.227 and 0.018, respectively. The blood/plasma partition coefficient (B:P) of both compounds was approximately at 0.6 unit.

### Development of PBPK models of DABE and its metabolites

In Step 1 of model development, the developed PBPK model of DABE linking DABE with all 3 metabolites was first evaluated by simulating the DDI between DABE and CTC. As shown in Supplementary Table S6, the model well described the magnitudes of changes in DAB plasma exposure in the DABE-CTC interaction studies following both microdose and therapeutic dose of DABE. However, in both scenarios, the plasma concentration-time profiles and PK parameters (C_max_ and AUC_0-inf_) of DAB were overpredicted (Supplementary Table S6; Supplementary Figure S3), suggesting the need for parameter optimization to capture the DAB profiles while maintaining the magnitudes of the DABE-CTC interaction.

In Step 2 of model development (Figure 1), the GSA using Morris’s method was applied to explore an additional mechanism to resemble the plasma DAB profile following microdose DABE administration (training dataset 1). The results suggested that the parameters related to BIBR0951 clearance (e.g., intrinsic clearance for biliary excretion, CL_int, bile_) had a remarkable influence on the DAB exposure (Supplementary Figure S4). After incorporating the estimated CL_int, bile_ of BIBR0951 at 273 μL/min/million cells, the models adequately captured the PK profile of DAB following microdose DABE (Supplementary Figure S5A, left and middle panels), but still overpredicted the DAB profile following DABE at the therapeutic dose (Supplementary Figure S5B, left and middle panels). To recover the DAB profile following therapeutic dose DABE, the input parameters related to formulation, aqueous solubility, and precipitation were included into the DABE model, as described in the “*Material and methods*” section. Based on model training set 3 (Blech et al., 2008), the CSR and PRC values were estimated at 17.9 unit and 2.88 h^−1^, respectively. After the incorporation of solubility/precipitation model and formulation of DABE, the models could reasonably predict the plasma DAB profiles following DABE administration at both micro- and therapeutic doses (Supplementary Figures S5A, B, right panel).

**FIGURE 3:**
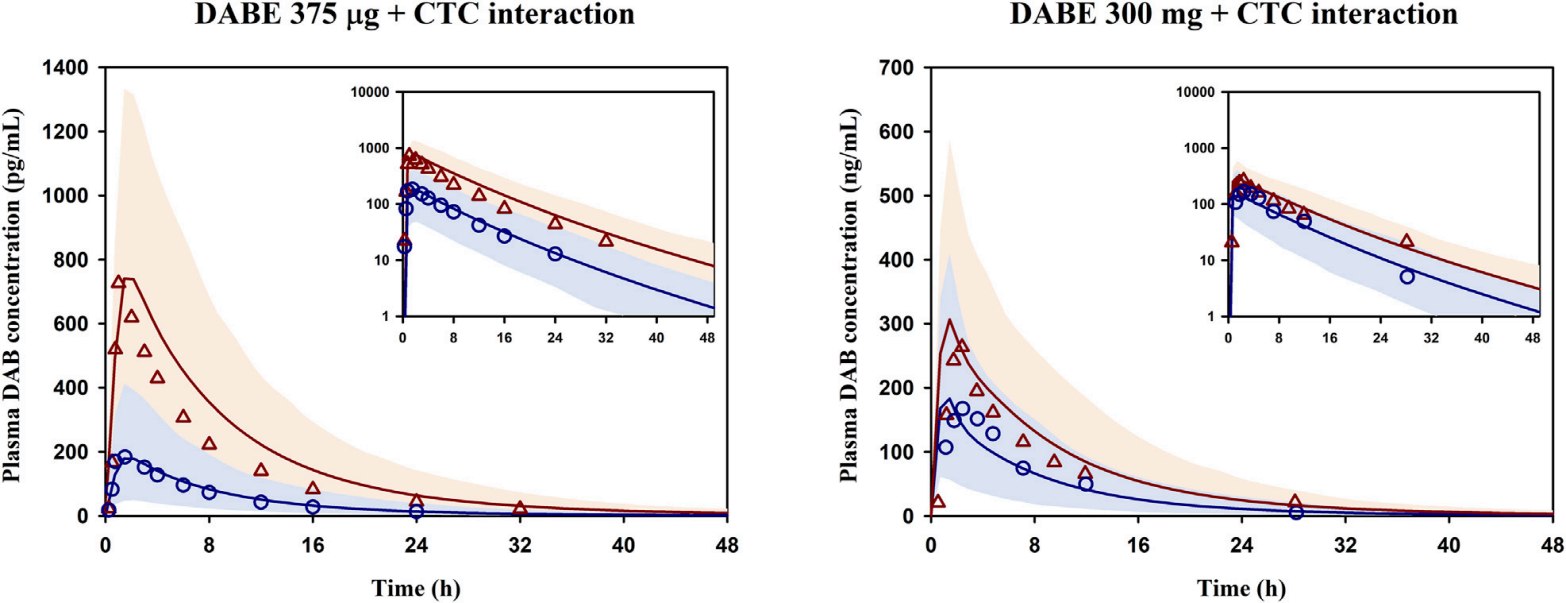
Simulated and observed mean plasma concentration-time profiles of DAB following DABE-CTC DDI evaluated at the microdose (375 μg, left panel) or therapeutic dose (300 mg, right panel) of DABE from Prueksaritanont et al. (2017) and Delavenne et al. (2013), respectively. Blue circles and blue lines represent the observed and simulated DAB levels following DABE alone, respectively. Red triangles and red lines represent the observed and simulated DAB levels following DABE-CTC coadministration, respectively. Shaded blue and red areas show the 95% confidence interval of the simulated DAB concentrations in the absence and presence of CTC, respectively.

In Step 3 of model development (Figure 1), the final PBPK model of DABE linking with its metabolites was then used for simulation of DDI between DABE and CTC again. As shown in Figure 3, the model well captured the observed plasma concentration-time profiles of DAB following microdose and therapeutic dose either in the presence or absence of CTC. Furthermore, the plasma PK parameters and DDI magnitudes were reasonably predicted within the acceptance ranges (Supplementary Table S7).

### Qualification of PBPK models of DABE and its metabolites

The developed PBPK model of DABE linked with its metabolites was then qualified against several independent datasets, including SD PK, MD PK, and DDI with CYP3A/P-gp inhibitors (Supplementary Table S1).

As shown in Supplementary Figure S6, the model well simulated the observed plasma concentration-time profiles of DAB following single and multiple dosing of DABE at both the microdose and therapeutic dose. In addition, the model could adequately predict the plasma PK parameters of DAB in various scenarios, except for qualification dataset 9 (Table 1). Noteworthy that the observed values in this dataset 9 are higher than the corresponding values in dataset 2 with the same 300 mg DABE dose, but in a comparable range to those reported in dataset 10–11 following 150 mg DABE dose. Overall, the GMFE values for predicting DAB C_max_ and AUC_0-inf_ were at 1.07 and 1.09, respectively, indicating successful predictions with negligible bias.

**TABLE 1:** Prediction of plasma PK parameters of DAB in various scenarios using the final PBPK models of DABE and its metabolites.

Set	DABE		C_max_ (ng/mL)			AUC_0-inf_ (ng·h/mL)			References
	Dose	Formulation	Observed	Simulated	Criteria^a^	Observed	Simulated	Criteria^a^	
Training sets									
1	375 µg	Solution with precipitation	0.17 (0.13–0.23)	0.15 (0.14–0.17)	0.09–0.34	1.44 (1.03–2.02)	1.44 (1.30–1.60)	0.72–2.88	Prueksaritanont et al. (2017)
2	300 mg	Solid IR	174^#^ (92–310)	166^#^ (40–618)	87–348	1,220^#^ (586–2,227)	1,181^#^ (280–4,538)	610–2,440	Delavenne et al. (2013)
3	200 mg	Solution with precipitation	145 ± 45^**#450">ǂ**^	151 ± 90^**#450">ǂ**^	96–218	930 ± 232^**#450">ǂ**^	1,166 ± 759^**#450">ǂ**^	668–1,295	Blech et al. (2008)
Qualification sets									
4	400 mg SD	Solution with precipitation	281	249	140–562	1,254	1,128	627–2,508	Stangier (2008)
	400 mg MD	Solution with precipitation	662	334	331–1,324	5,071	2,805	2,535–10,142	Stangier (2008)
5^ψ^	150 mg	Solid IR	107 ± 72^**#450">ǂ**^	124 ± 80	47–244	937 ± 649^**#450">ǂ**^	965 ± 655^**#450">ǂ**^	403–2,178	Stangier et al. (2008)
6	750 µg	Solution with precipitation	0.38 (0.30–0.47)	0.33 (0.30–0.36)	0.19–0.76	3.11 (2.58–3.76)	3.10 (2.79–3.44)	1.56–6.22	Rattanacheeworn et al. (2021)
9	300 mg	Solid IR	83 (66)	170 (61)	60–116	547 (77)	1,239 (68)	376–796	Gouin-Thibault et al. (2017)
10,11	150 mg	Solid IR	63 (122)	105 (64)	25–156	536 (110)	793 (68)	229–1,252	Hartter et al. (2013)

Data were reported as geometric means (90% CI, or %CV), except ^**#**^median (min-max) and ^ǂ^arithmetic mean ± s.d. ^ψ^ Data were from total DAB, levels (free + conjugated).

^a^ Acceptance range was defined based on alternative success criteria (Abduljalil et al., 2014).

**FIGURE 4:**
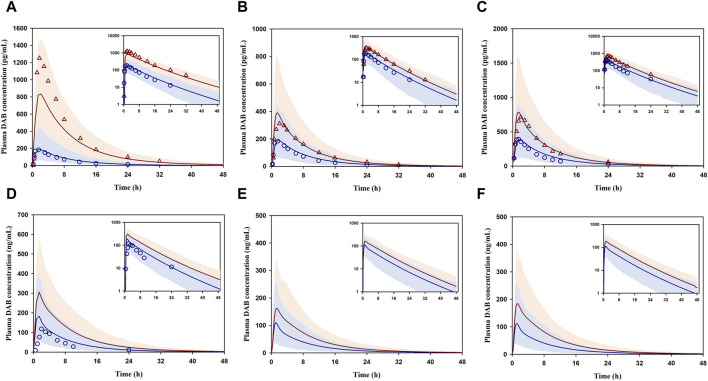
DDI prediction between DABE and CYP3A/P-gp inhibitors: ITZ **(A)**, RF **(B,C)**, CTC **(D)**, VP concomitantly **(E)**, and VP 1 h before DABE **(F)** from Prueksaritanont et al. (2017), Rattanacheeworn et al. (2021), Gouin-Thibault et al. (2017), and Hartter et al. (2013), respectively. Blue circles and blue lines represent the observed and simulated DAB levels for the control phase, respectively. Red triangles and red lines represent the observed and simulated DAB levels for the interaction phase, respectively. Shaded blue and red areas are the 95% confidence interval of the simulated DAB concentrations in the absence and presence of perpetrators, respectively.

For DDI prediction, the developed model was able to reasonably simulate the plasma concentration-time profiles of DAB following coadministration of DABE and various CYP3A/P-gp inhibitors (Figures 4A–F). Notably, there have been no reports on the plasma DAB profiles of unconjugated DAB for the data set 9–11 (Figures 4D–F); thus, we could not evaluate the simulated profiles using the VPC method. In addition, the DDI magnitudes (C_max_ and AUC_0-inf_ ratio) following DABE administration at the microdose and therapeutic dose were all well predicted within the acceptance ranges (Table 2). The GMFE values for predicting C_max_ and AUC_0-inf_ ratio were at 0.88 and 0.87, respectively, suggesting that the model can accurately predict DDI magnitudes with minimal bias.

**TABLE 2:** Prediction of DDI between DABE and CYP3A/P-gp inhibitors using the final PBPK models of DABE and its metabolites.

Set	DABE		Perpetrators	C_max_ ratio			AUC_0-inf_ ratio			References
	Dose	Formulation		Observed	Simulated	Criteria^a^	Observed	Simulated	Criteria^a^	
Training sets										
1	375 µg	Solution with precipitation	CTC 500 mg PO BID 5 days	4.57 (2.85–7.34)	4.46 (4.17–4.78)	2.57–8.14	4.02 (2.99–5.41)	4.64 (4.33–4.97)	2.30–7.04	Prueksaritanont et al. (2017)
2	300 mg	Solid IR	CTC 500 mg PO BID 5 days	1.60^#^	1.61^#^	1.16–2.20	1.49^#^	1.97^#^	1.12–1.98	Delavenne et al. (2013)
Qualification sets										
6	750 µg	Solution with precipitation	RF 600 mg PO SD	1.86 (1.43–2.43)	2.16 (2.08–2.23)	1.27–2.72	2.22 (1.74–2.83)	1.94 (1.88–1.99)	1.49–3.68	Rattanacheeworn et al. (2021)
7	375 µg	Solution with precipitation	ITZ 200 mg (solution) PO QD 5 days	6.42 (4.57–9.01)	4.82 (4.48–5.19)	3.48–11.84	6.92 (4.96–9.66)	5.12 (4.74–5.52)	3.73–12.84	Prueksaritanont et al. (2017)
8	375 µg	Solution *w*th precipitation	RF 600 mg PO SD	1.78 (1.47–2.16)	2.16 (2.09–2.24)	1.24–2.56	2.32 (1.86–2.90)	1.94 (1.89–1.99)	1.51–3.76	Prueksaritanont et al. (2017)
9	300 mg	Solid IR	CTC 500 mg PO BID 5 days	1.71	1.61	1.21–2.42	1.97	1.97	1.32–2.94	Gouin-Thibault et al. (2017)
10	150 mg	Solid IR	VP IR 120 mg PO SD	2.15	1.55	1.40–3.30	1.98	1.88	1.32–2.96	Hartter et al. (2013)
11	150 mg	Solid IR	VP IR 120 mg PO SD 1 h before DABE	2.70	1.74	1.66–4.40	2.37	2.07	1.50–3.74	Hartter et al. (2013)

Data were reported as geometric means (90% confidence interval or % coefficient of variation), except. ^**#**^median.

^a^ Acceptance range was defined based on Guest’s DDI, prediction criteria (Guest et al., 2011).

Taken together, the results suggested that the comprehensively developed PBPK model of DABE with its metabolites adequately predicted the plasma PK profiles and parameters of DAB after oral administration of DABE with and without CYP3A/P-gp inhibitors. Therefore, the model was sufficiently qualified for further applications.

### Investigation of the disparity in DDI magnitudes of DABE-CTC interaction following 2 different dose levels of DABE

**FIGURE 5:**
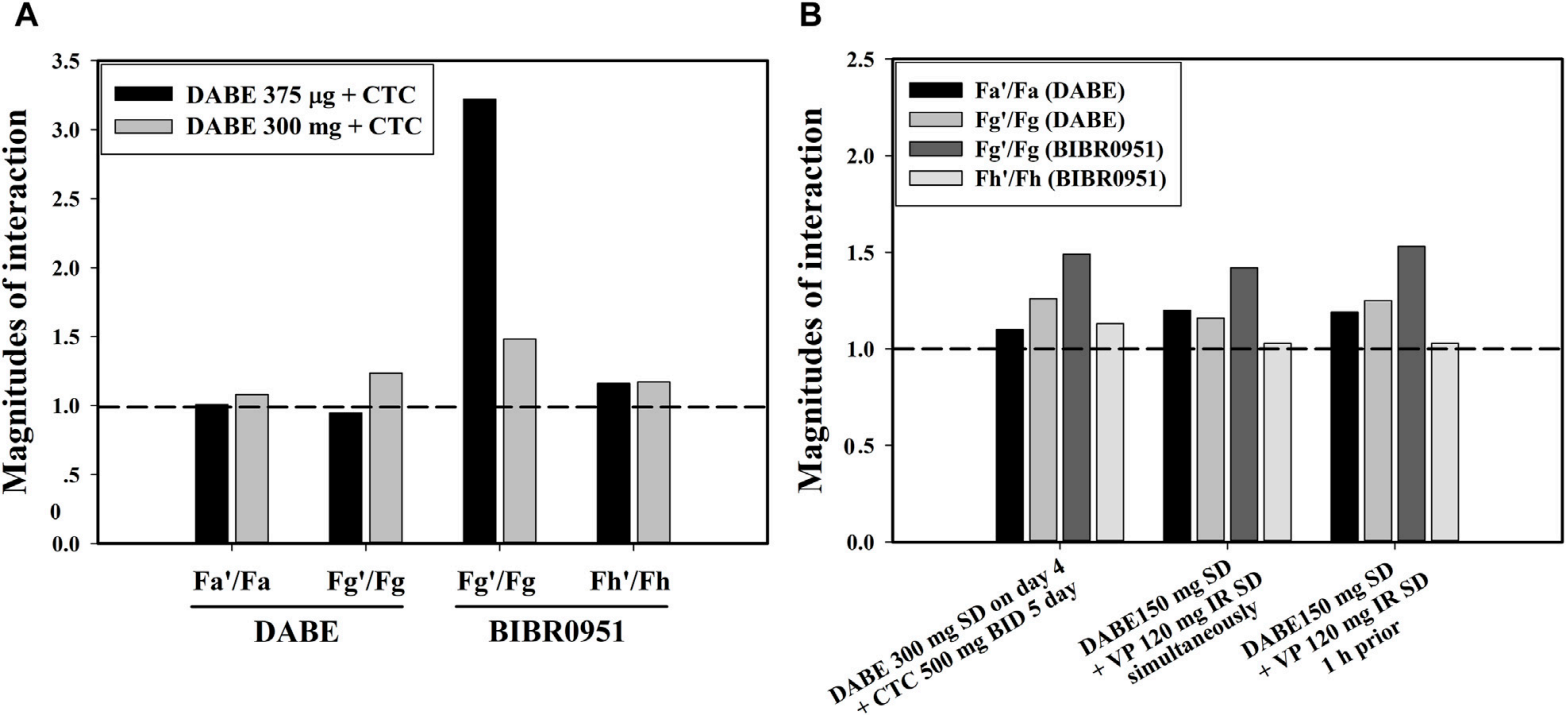
**(A)** The inhibitory effects of CTC on the intestinal P-gp and gut/hepatic CYP3A following a microdose (375 µg) and therapeutic dose (300 mg) of DABE. **(B)** The inhibitory effects of CTC and VP simultaneously with or 1 h prior to DABE) on the intestinal P-gp and gut/hepatic CYP3A following therapeutic doses (300 mg or 150 mg) of DABE. The presystemic parameters including fraction absorbed (Fa), fraction escaping gut metabolism (F_g_), and fraction escaping hepatic metabolism (F_h_) were computed from the simulation outputs. The parameters with and without accent (’) denote the inhibited and control conditions, respectively.

The qualified PBPK model of DABE with a link to its metabolites was subsequently applied to gain a mechanistic understanding of the DDI magnitude disparity of the DABE-CTC interaction observed following administration of DABE at the microdose (375 µg) versus therapeutic dose (300 mg). The simulated results of the DABE-CTC interaction (Figure 3) were dissected for the inhibitory effects of CTC on the intestinal P-gp and gut/hepatic CYP3A. When comparing the DABE-CTC interaction using the microdose and therapeutic dose of DABE, there was a remarkable difference in the F_g_’/F_g_ ratio (∼3.3 vs. 1.5) of the gut metabolite BIBR0951 (Figure 5A). On the contrary, either small or no difference was observed for other presystemic PK parameters (F_a_’/F_a_ and F_g_’/F_g_ of DABE, and F_h_’/F_h_ of BIBR0951) (Figure 5A). These results indicated that the disparity in the magnitudes of the DABE-CTC interaction observed between two doses of DABE was driven mainly by the intestinal CYP3A-mediated oxidative metabolism of BIBR0951, with a minimal contribution from the gut P-gp-mediated efflux of DABE or any other presystemic events. The higher magnitude of F_g_’/F_g_ ratio at the micro-*vs* therapeutic dose is consistent with the *in vitro* report that the CYP3A-mediated oxidation was saturable (Udomnilobol et al., 2023). Interestingly, following the therapeutic dose of DABE with CTC, the intestinal CYP3A-mediated oxidation of BIBR0951 also played a significant, albeit relatively small role, with the F_g_’/F_g_ ratio for BIBR0951 being higher than any other ratios shown in Figure 5A.

### Mechanistic insights into the relative significance of the intestinal P-gp- and CYP3A-mediated pathways to DDI magnitudes of DABE-VP interaction

The qualified model was also used to dissect the inhibitory effects of VP on the intestinal P-gp and gut/hepatic CYP3A for the clinical DABE-VP interaction results (Table 2 dataset 10–11) following administration of DABE at the therapeutic dose (150 mg). Based on the inhibitor model input parameters, VP is much more potent than CTC as a P-gp inhibitor (Supplementary Table S5), and thus could serve as a better tool for assessing the potential role of the gut P-gp in the intestinal absorption of DABE. Consistent with this, VP appeared to have a slightly higher impact than CTC on the gut P-gp, with F_a_’/F_a_ ratio of 1.2 versus 1.05 for CTC (Figure 5B). However, the relative impact of the gut P-gp-mediated efflux of DABE was still rather limited, when compared with the intestinal CYP3A-mediated oxidation of BIBR0951 (F_g_’/F_g_ ratio of 1.5) (Figure 5B).

### Investigation of nonlinearity in DAB exposure following oral ascending doses of DABE

**FIGURE 6:**
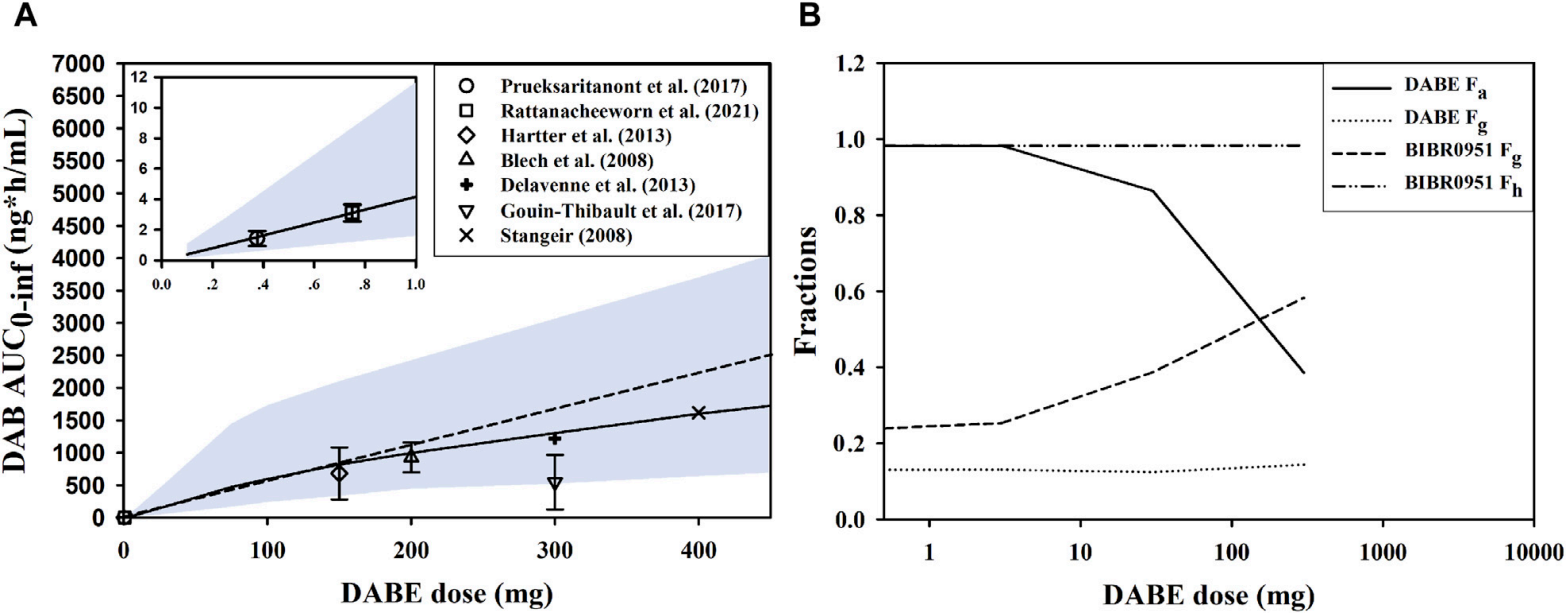
Investigation of nonlinearity in plasma DAB exposure. **(A)** Simulated and observed AUC_0-inf_ of DAB following single oral administration of DABE from 0.1 to 400 mg doses, and from 0.1 to 1.0 mg doses (see insert). The symbols and solid lines represent the observed and simulated data, respectively. The dashed line represents proportionality between AUC0-inf vs. DABE doses. The symbols and lines represent the observed and simulated data, respectively. The shaded areas are the 95% confidence interval of the simulated data. **(B)** The simulated presystemic parameters, including fraction absorbed (F_a_), fraction escaping gut metabolism (F_g_), and fraction escaping hepatic metabolism (F_h_) following DABE doses at 0.3, 3, 30, and 300 mg.

We first simulated the plasma DAB profiles over a wide dose range of 0.1–400 mg following single oral administration of DABE. When compared with the observed data, the model could reasonably predict the systemic exposure of DAB throughout the dose range employed. Based on the simulation outputs, the AUC_0-inf_ values of DAB were approximately dose-proportional from 75 to 400 mg DABE. When the dose of DABE was <75 mg (lower than therapeutic doses), the plasma DAB exposure was slightly over 2-fold lower than that extrapolated from the therapeutic dose range of DABE (Figure 6A).

To gain an insight into the underlying mechanisms of nonlinearity, the presystemic PK parameters were computed from the simulation outputs. By increasing the doses of DABE from 0.3 to 300 mg, there was a dramatic decrease in the F_a_ of parent DABE from approximately 1.0 to 0.4 (Figure 6B), consistent with its poor solubility. The decreases in F_a_ with increasing doses were not supportive of the gut P-gp-mediated DABE efflux being a major contributing factor (versus the solubility factor) in the overall intestinal absorption of DABE across dose levels. On the other hand, the F_g_ of BIBR0951 was increased following the ascending doses of DABE; this was in line with the *in vitro* result of the saturable CYP3A-mediated metabolism of BIBR0951 being the sole metabolic pathway mediating the gut metabolism of BIBR0951 (Udomnilubol et al., 2023). Noteworthy that the minimal changes in the F_g_ of DABE or F_h_ of BIBR0951 across different doses of DABE also are not inconsistent with the earlier *in vitro* findings that CYP3A contributed only partly to the metabolism at the gut and liver of DABE and BIBR0951, respectively (Udomnilubol et al., 2023). Taken together, the simulated results showed that both the solubility-limited DABE absorption and the saturation of intestinal CYP3A-mediated BIBR0951 oxidation were the two major contributing factors, opposing to each other, to the somewhat modest nonlinearity observed in the plasma DAB exposure.

## Discussion

In this study, a comprehensive mechanistic PBPK model linking DABE and its metabolites (BIBR0951, BIBR1087, and DAB) was successfully built using a middle out approach with all relevant disposition pathways known to-date, including solubility/permeability and formulation of DABE, gut P-gp mediated DABE efflux, and CYP3A4/5-mediated oxidative metabolisms of DABE and BIBR0951. The developed model adequately predicted the plasma PK of DAB in several scenarios, i.e., SD/MD PK and DDIs between DABE and CYP3A/P-gp perpetrators. Furthermore, the model also provided mechanistic understandings of the relative contribution of the intestinal P-gp-mediated efflux versus CYP3A-mediated metabolism in the disposition of DABE and its clinical DDIs with P-gp/CYP3A inhibitors following administration of DABE at the micro- and therapeutic doses.

To date, several semi-mechanistic PBPK models of DABE and DAB have been successfully developed for various applications, such as DDI prediction (Zhao and Hu, 2014; Doki et al., 2019; Yamazaki et al., 2019; Wang et al., 2020; Lang et al., 2021), formulation development (Farhan et al., 2021), and PK prediction in renally-impaired patients (Doki et al., 2019; Moj et al., 2019). However, none of these have considered or incorporated the two intermediate metabolites (BIBR0951 and BIBR1087) into their PBPK models. Additionally, the presystemic DAB formation in the previously published models was achieved by a direct hydrolysis from DABE, which should be considered as “black box” modeling. Consequently, the additional DDI mechanisms (e.g., gut and hepatic CYP3A-mediated oxidation) could not be incorporated into those models and therefore their potential role in DDI assessment could not be investigated. Furthermore, our earlier attempts to construct a PBPK model of DABE and its metabolites without incorporating CYP3A-mediated pathways failed to capture the over twofold difference in the DDI magnitudes of the DABE-CTC interaction observed between two dose levels of DABE (data not shown). Interestingly, the model developed by Lang et al. (2021), in which the CYP3A related mechanisms were not incorporated, also showed an over-prediction of the DAB PK (C_max_ and AUC) in both studies with the therapeutic dose of DABE (study set 2 and 9 in Table 1), despite the DDI magnitudes met their prediction acceptance criteria.

Following microdose administration of DABE, the magnitude of the DABE-CTC interaction was ≥ twofold higher than that observed following administration of the therapeutic dose. Prueksaritanont et al. (2017) proposed that the disparity in the DDI magnitudes of the DABE-CTC interaction could be from 1) a partial saturation of intestinal P-gp; and 2) a potential involvement of CYP3A in the disposition of microdose DABE. A recent publication using the PBPK approach suggested that CTC-mediated P-gp inhibition at different intestinal regions could explain the differences in the DDI magnitudes between microdose and therapeutic dose DABE (Lang et al., 2021). Alternatively, our recent *in vitro* findings demonstrated that when the concentrations of both compounds were <10 µM (well below the theoretical gut concentration following therapeutic dose of DABE), CYP3A may be significantly involved in the presystemic metabolism of both DABE and gut metabolite BIBR0951, (Udomnilobol et al., 2023). In this PBPK study, we highlighted that the intestinal CYP3A-mediated oxidative metabolism of the gut metabolite BIBR0951, and not the gut P-gp-mediated efflux nor the CYP3A-mediated gut metabolism of DABE, played a major role in the disparity of the DDI magnitudes of the DABE-CTC interaction observed at two dose levels of DABE.

Unexpectedly, the results showing greater magnitude of Fg’/Fg ratio above 1 (Figure 5B) also suggested that the CYP3A-mediated gut metabolism, particularly of the intermediate metabolite BIBR0951 contributed partly to the DDI magnitudes observed following the administration of therapeutic dose DABE with CTC or VP, another CYP3A/P-gp inhibitor. Additionally, at the therapeutic dose of DABE, the impact of the intestinal P-gp-mediated DABE efflux on the DDI magnitudes appeared to be limited, even with the relatively potent P-gp inhibitor VP. In this regard, it is worth pointing out that the P-gp Ki values of VP and its metabolite used in our study were >20-fold lower than those employed in the Lang et al. (2021) study, and despite this fact, we were unable to see much of the VP impact on the P-gp efflux (Figure 5B). Additionally, attempts to increase the P-gp Jmax value of DABE by 2-fold, which was the highest value permissible by our model qualification criteria, did not significantly increase the impact of gut P-gp vs. CYP3A4/5 (data not shown). Although unexpected and in contrast to the widely accepted notion that DABE is a selective gut P-gp probe substrate, our results can be rationalized as follows. According to the biopharmaceutical classification system (BCS), DABE is categorized as a BCS class II compound exhibiting low solubility and high permeability properties (FDA, 2010b). Following the microdose DABE administration (375–750 µg) when its solubility was not limited, DABE might highly permeate across the intestinal epithelium, and thus, the impact of the P-gp efflux under this condition was minimal. When DABE was administered at the therapeutic dose (75–300 mg), the luminal concentration of DABE at the compartment of jejunum I was predicted to be in a mM level, far exceeding the P-gp K_m_ used in this study (2.6 µM). Even considering its limited solubility, the luminal concentration could still reach this Km value, and therefore it is conceivable that the intestinal P-gp-mediated DABE efflux would have a limited role following DABE at the therapeutic dose.

Apart from the underlying mechanism of the DABE-CTC interaction, our PBPK models also shed some light on the potential causes of the nonlinearity in plasma DAB exposure. Following the microdose DABE, the systemic exposure of DAB was approximately 2-fold lower than expected based on the linear dose-exposure relationship established from the therapeutic dose range (Stangier et al., 2007; Prueksaritanont et al., 2017). Our simulation results suggested that the nonlinearity of DAB exposure was possibly due to the solubility-limited DABE absorption (F_a_ of DABE) and the saturation of CYP3A-mediated BIBR0951 oxidation (F_g_ of BIBR0951), both of which occurred at the gut level and thus an oral-dosing specific finding. The opposing trend between the F_a_ and F_g_ moderated the extent of decreases in the exposure of DAB, resulting in only about 2-fold deviation from dose-exposure linearity despite the large dose difference between the micro- and therapeutic doses of oral DABE. Conceivably, this PK non-linearity issue would be less a concern following DABE intravenous (IV) administration. In this regard and considering that DABE is a relatively narrow therapeutic index drug, a microdose DABE could remain a possible clinical tool, when given IV and especially for safe DDI assessments pertaining primarily to hepatic CYP3A.

It is worth pointing out that although the absolute extent of CYP3A4/5-mediated metabolism was higher for DABE compared to BIBR0951 (Supplementary Tables S2, S3), the intestinal first-pass effect of BIBR0951 was found to play a more significant role than that of DABE in mediating the DDI (Figure 5) and the PK non-linearity observed across DABE dose range (Figure 6B). This observation could potentially be attributable to the fact that for DABE, the CYP3A4/5-mediated intestinal metabolism was a relatively minor pathway versus the CES-mediated hydrolysis, while for BIBR0951, it was the sole metabolic pathway (Udomnilobol et al., 2023).

Nevertheless, our PBPK models had several limitations worth highlighting. First, the metabolism of DAB via the glucuronidation pathway, a minor pathway (Blech et al., 2008), was not included, thus the models could not simulate the plasma PK of total DAB (free + glucuronide conjugated forms). Second, despite a very limited dataset for both the intermediates available for comparison with the simulation results, the models appeared to dramatically overpredict the plasma levels of the two intermediate metabolites (BIBR0951 and BIBR1087) (Supplementary Figures S7A, B). We hypothesized that this apparent overprediction was possibly because of the unavailability of the known disposition pathways as well as the true primary PK parameters (V_d,ss_ and CL_sys_) of both compounds. This reason is supported by the finding that the developed model appeared to be able to capture plasma levels, albeit there was limited availability, of the parent DABE (Supplementary Figure S7C). The developed model could also reasonably predict (within 2-fold error of the observed data) the PK parameters of DABE (C_max_ 2.10 ng/mL; AUC_0-1.5h_ 2.03 ng·h/mL) (C_max_ 2.90 ng/mL; AUC_0-1.5h_ 2.81 ng·h/mL) (Stangier et al., 2008). Lastly, our qualification set did not include a DDI study of DABE with a specific inhibitor of P-gp as all the inhibitors used in the study were considered dual CYP3A/P-gp inhibitors. We have not been able to find such a study in the literature to either corroborate or refute our findings.

In conclusion, the comprehensive mechanistic PPBK model of DABE with its intermediate metabolites connected to its pharmacologically active species DAB was successfully developed using a middle-out approach, with comprehensive *in vitro* and observed clinical datasets available to date. As an alternative to other publicly available models, our PBPK model could provide mechanistic insights into the CYP3A-related disposition mechanisms of DABE and its metabolites at the presystemic level. In addition, our model suggested that consistent with the *in vitro* findings, the involvement of CYP3A in the DDIs with CYP3A/P-gp inhibitors was much greater following administration of DABE at the microdose than at the therapeutic dose. In contrast, the contribution of the gut P-gp in the DDIs was negligible following the microdose and became apparent, despite small in magnitude only after the therapeutic dose and with a relatively potent P-gp inhibitor. The potentially limited role of gut P-gp-mediated efflux and the apparently appreciable contribution of gut CYP3A even following administration of a therapeutic dose of DABE suggest a possible overestimation of the gut P-gp contribution when using DABE as a clinical probe in the DDI assessment with a dual CYP3A/P-gp inhibitor. Overall, this study highlighted the potential for DABE being a non-selective gut P-gp probe substrate for assessing clinical DDIs with dual CYP3A/P-gp inhibitors, regardless of DABE dose levels. Additionally, the results hint at a need for careful consideration and result interpretation/extrapolation when considering and applying a microdose approach in clinical DDI assessments and also in first-in-human (Phase 0) PK characterization, especially for an orally administered compound with limited bridging absorption and disposition information across the intended dose range.

## Data Availability

The original contributions presented in the study are included in the article/Supplementary Material, further inquiries can be directed to the corresponding authors.

## Abbreviations

BIBR0951, dabigatran ethylester; BIBR1087, desethyl dabigatran etexilate; CES, carboxylesterase; CTC, clarithromycin; CYP, cytochrome P450; DAB, dabigatran; DABE, dabigatran etexilate; DDI, drug-drug interaction; ITZ, itraconazole; PBPK, physiologically based pharmacokinetics; P-gp, P-glycoprotein; RF, rifampicin; VP, verapamil.
