# Supplementary material for: Assessing the relative contribution of CYP3A-and P-gp-mediated pathways to the overall disposition and drug-drug interaction of dabigatran etexilate using a comprehensive mechanistic physiological-based pharmacokinetic model
Source: Front Pharmacol. 2024 Mar 7;15:1356273. doi: 10.3389/fphar.2024.1356273 (PMC10955231; doi:10.3389/fphar.2024.1356273)
Supplement: Supplementary file 1 [file DataSheet1.pdf]

Supplementary Material

Assessing the relative contribution of CYP3A- and P-gp-mediated pathways to the overall disposition and drug-drug interaction of dabigatran etexilate using a comprehensive mechanistic physiological-based pharmacokinetic model

Udomsak Udomnilobol[1], Wilasinee Dunkoksung[1], Watchara Sakares[2], Suree Jianmongkol[2*], Thomayant Prueksaritanont[1*]

[1]Chulalongkorn University Drug Discovery and Drug Development Research Center (Chula4DR), Chulalongkorn University, Bangkok 10330, Thailand

[2]Department of Pharmacology and Physiology, Faculty of Pharmaceutical Sciences, Chulalongkorn University, Bangkok 10330, Thailand

*** Correspondence:**

Corresponding author: T. P. : thomayant.p@pharm.chula.ac.th

Co-corresponding author: S. J : suree.j@pharm.chula.ac.th

1 Supplementary Methods

Materials

All materials used in this study were the highest grade available commercially. Dabigatran etexilate (DABE), desethyl dabigatran etexilate (BIBR1087), dabigatran ethylester (BIBR0951), and dabigatran (DAB) were purchased from Toronto Research Chemical Inc. (Ontario, Canada). Acetonitrile was purchased from Honeywell Burdick and Jackson (Fischer Scientific, MI, USA). Chloroquine diphosphate, formic acid, ketoconazole, labetalol hydrochloride, and phenylmethylsulfonyl fluoride (PMSF) were purchased from Tokyo Chemical Industry Co., Ltd. (Chuo-ku, Tokyo, Japan). Pooled human liver microsome (HLM) was purchased from Gibco Life Technologies (Thermo Fischer Scientific Inc., MA, USA). Pooled human intestinal microsome (HIM) was purchased from Corning (Corning Incorporated, NY, USA). Pooled human plasma was purchased from Innovative Research, Inc. (Novi, MI, USA). Rapid equilibrium dialysis (RED) device single-used plate with inserts of 8K MWCO was purchased from Thermo Scientific (Thermo Fischer Scientific Inc., MA, USA).

Plasma stability

Fresh human plasma (Thai, male, age of 30-40 years) was incubated with BIBR0951 or BIBR1087 (1 or 100 μM) at 37°C up to 2 h. The organic solvent (acetonitrile) in the system was kept not more than 1%. After incubation, the samples were quenched with 3 volumes of ice-cold acetonitrile + 0.1 % formic acid containing labetalol (internal standard). After centrifugation, the supernatants were collected and diluted with water + 0.1% formic acid before LC-MS/MS analysis.

In vitro enzyme kinetic

Kinetic parameters of CES-mediated BIBR1087 hydrolysis were determined in HLM and HIM under linear metabolite formation condition. The experiments were conducted as described previously (Udomnilobol et al., 2023). The Michaelis-Menten constants (K_m) and maximum velocity (V_{max}) were estimated from a graph plotted between rates of metabolites formation versus substrate concentrations, using the enzyme kinetic module in SigmaPlot software (version 13.0; SyStat Software, Inc., CA, USA).

In vitro-to-*in vivo* extrapolation factors

CYP3A4/5 pathways

Intersystem extrapolation factors (ISEF) of intrinsic clearance (CL_{int}) was calculated, using the following equation (Proctor et al.,2004):

$$\mathrm{ISEF} = \frac{\mathrm{CL_{int}\ (HLM)}}{\mathrm{CL_{int}\ (rhCYP) \times CYP\ abundance\ (HLM)}}$$

where CL_{int} (HLM) is HLM intrinsic clearance, CL_{int} (rhCYP) is recombinant human CYP3A4/5 intrinsic clearance of DABE, BIBR095, and CYP abundance (HLM) is an abundance of CYP3A4/5 in the liver. All *in vitro* kinetic parameters, specifically Km and Vmax of DABE and its metabolites, were obtained from Udomnilobol et al. (2023) . Using the Simcyp population based ADME simulator software version 20 (Certara, Sheffield, UK), the *in vitro* Vmax values were scaled up to whole liver Vmax values through multiplying with ISEF, liver CYP3A4/5 abundance, amount of microsomal protein per liver, and liver weight. Then the well-stirred model was used for extrapolating organ CL to in *vivo* CL values.

CES1/2 pathways

The *in vitro* values of Km and Vmax (HLM and HIM) were obtained from Udomnilobol et al. (2023). Through the software, the well-stirred model was applied to obtain *in vivo* CL values upon conversion of *in vitro* Vmax to whole liver Vmax values (by multiplying with the amount of microsomal protein per liver and liver weight for HLM and the amount of microsomal protein per intestine).

Plasma esterase

In vivo clearance from plasma esterase was estimated from the *in vitro* half-life values of DABE and BIBR0951, using the equation derived from Imai et al., 2022 in the Simcyp software:

$$\mathrm{Plasma\ clearance} = \frac{\mathrm{In2}}{\mathrm{half-life}} \times \mathrm{Volume\ of\ Plasma}$$

Rapid equilibrium dialysis

Fractions unbound in plasma ($f_{u,p}$) of BIBR0951 and BIBR1087 were determined by rapid equilibrium dialysis method. Briefly, the compound at 1 µM in pooled human plasma was dialyzed against phosphate buffer saline (0.1 M sodium phosphate + 150 mM NaCl, pH 7.40) at 37°C in a humidified CO_2 incubator for 4 h. The organic solvent in the system was kept not more than 1%. For inhibition of BIBR0951 and BIBR1087 hydrolysis in plasma, the experiment was performed in the presence of esterase inhibitor, 1 mM phenylmethylsulfonyl

fluoride (PMSF). After incubation, the dialyzed plasma and buffer were collected and added with an equal volume of blank buffer and plasma, respectively. Then, the samples were quenched with 3 volumes of ice-cold acetonitrile + 0.1 % formic acid containing labetalol as an internal standard. After centrifugation, the supernatants were collected and diluted with water + 0.1% formic acid prior to LC-MS/MS analysis. The $f_{u,p}$ was calculated by dividing the peak area ratio in the dialyzed buffer to that in the dialyzed plasma. The % recovery was calculated by 100 × (a summation of peak area ratio in the dialyzed buffer and plasma)/peak area ratio in the initial plasma before dialysis. In this experiment, ketoconazole (1 μM) was used as a positive control.

Blood/plasma partitioning

Blood to plasma partition coefficients (B:P) of BIBR0951 and BIBR1087 were determined as described previously (Yu et al., 2005). Briefly, the compounds were separately incubated at 1 μM in whole blood and corresponding plasma (reference plasma) at 37°C for 1 h. The organic solvent in the incubations was kept not more than 1%. For inhibition of BIBR0951 and BIBR1087 hydrolysis in plasma, the experiment was performed in the presence of esterase inhibitor, 1 mM phenylmethylsulfonyl fluoride (PMSF). At 0 and 1 h, both whole blood and reference plasma were collected and centrifuged at 2,000 g for 10 min at 25°C. Then, plasma samples were collected from the centrifuged whole blood and reference plasma, and immediately quenched with ice-cold acetonitrile + 0.1 % formic acid containing internal standard (labetalol). After centrifugation, the supernatants were collected and diluted with water + 0.1% formic acid prior to LC-MS/MS analysis. The B:P values were calculated by dividing the peak area ratio in the partitioned plasma (from whole blood) at 1 h to that in the reference plasma at 1 h. The % recovery was calculated from the formula: 100 × (peak area ratio in the reference plasma at 1 h/peak area ratio in the reference plasma at 0 h). In this experiment, chloroquine (5 μM) was used as a positive control.

Quantitative LC-MS/MS bioanalysis

DABE and its metabolites in samples were analyzed by AB SCIEX QTRAP6500+ LC-MS/MS system. The chromatographic separation was performed by Exion LC AD100 system coupled with Phenomenex Kinetex® C-18, 1.7 µm, 50×2.1 mm analytical column. The mobile phase was run at 0.4 mL/min using a binary system of water + 0.1% formic acid (phase A) and acetonitrile + 0.1% formic acid (phase B). A gradient program was set as equilibration at 5%

B for 0.5 min, linear gradient 5-50% B for 3.5 min, ramping to 95% B for 1 min, holding at 95% B for 1 min, column reconditioning to 5% B for 0.2 min, and post-analysis equilibration at 5% B for 0.8 min. Turbo V^{TM} ion source was operated in the electrospray positive ionization mode under 40 psi of curtain gas, 4500 volts of ion spray voltage, 450°C of source temperature, and 40 psi for both heating and nebulizing gases. Data were acquired by multiple reaction monitoring (MRM) mode and processed by Analyst version 1.6 and MultiQuant version 3.0 softwares (AB SCIEX, MA, USA).

2 Supplementary Tables

Supplementary Table 1: Data sets for model development and qualification

Set	Subjects			DABE		Scenarios	Plasma profiles	References
	Age	n	Female ratio	Dose	Formulation			
**Training sets**								
1	19-55	12	?	375 µg	Solution	CTC 500 mg PO BID 5 days, DABE PO SD on day 4	Free DAB	(Prueksaritanont et al., 2017)
2	18-35	10	0	300 mg	Capsule	CTC 500 mg PO BID 5 days, DABE PO SD on day 4	Free DAB	(Delavenne et al., 2013)
3	39.9	10	0	200 mg	Solution	DABE PO SD	Free DAB	(Blech et al., 2008)
**Qualification sets**								
4	18-45	8	0	400 mg	Solution	DABE PO TID 6 days	Free DAB	(Stangier, 2008)
5	18-75	12	0.42	150 mg	Capsule	DABE PO SD	DABE, BIBR0951, BIBR1087, Total DAB	(Stangier et al., 2008)
6	20-40	20	0.60	750 µg	Powder	DABE PO SD + RF 600 mg PO SD	Free DAB	(Rattanacheeworn et al., 2021)
7	19-55	12	?	375 µg	Solution	ITZ 200 mg (solution) PO QD 5 days, DABE PO SD on day 4	Free DAB	(Prueksaritanont et al., 2017)
8	19-55	12	?	375 µg	Solution	DABE PO SD + RF 600 mg PO SD	Free DAB	(Prueksaritanont et al., 2017)
9	18-45	60	0	300 mg	Capsule	CTC 500 mg PO BID 5 days, DABE PO SD on day 4	Free DAB	(Gouin-Thibault et al., 2017)
10	18-55	20	0.40	150 mg	Capsule	DABE PO SD + VP IR 120 mg PO SD	Free DAB	(Hartter et al., 2013)
11	18-55	20	0.40	150 mg	Capsule	DABE PO SD at 1 h after VP IR 120 mg PO SD	Free DAB	(Hartter et al., 2013)

Supplementary Table 2: Input parameters of DABE model.

Parameters	DABE	
	**Values**	**Remarks**
**Physicochemical**		
MW (g/mol)	627.75	(FDA, 2010)
Log P	3.80	(FDA, 2010)
Compound type	Diprotic base	(Doki et al., 2019)
pKa1 / pKa2	4.0 / 6.7	(FDA, 2010)
B:P	1.26	(Doki et al., 2019)
$f_{u,p}$	0.063	(Doki et al., 2019)
**Absorption**		
Model	ADAM	
$P_{app,caco-2}$ ($\times10^{-6}$ cm/sec)	28.8	(Ishiguro et al., 2014)
Formulation type	Solution with precipitation or solid IR	(Farhan et al., 2021)
Solubility model	DLM	(Farhan et al., 2021)
Aqueous solubility (mg/mL) at pH 7.4	0.003	(FDA, 2010)
Precipitation model	First order (Model 2)	(Farhan et al., 2021)
CSR	17.9	Estimated
PRC (1/h)	2.88	Estimated
**Distribution**		
Model	Full PBPK (Method 2)	
$V_{d,ss}$ (L/kg)	15.08	Predicted
**Elimination**		
Model	Enzyme kinetic	
Pathway 1 (oxidative metabolism)		
CYP3A4 V_{max} (pmol/min/pmol CYP)	59.7	(Udomnilobol et al., 2023)
K_m (µM)	1.4	(Udomnilobol et al., 2023)
ISEF	0.3	Calculated
CYP3A5 V_{max} (pmol/min/pmol CYP)	68.3	(Udomnilobol et al., 2023)
K_m (µM)	1.1	(Udomnilobol et al., 2023)
ISEF	0.3	Calculated
Pathway 2 (DABE to BIBR0951)		
CES1 V_{max} (pmol/min/mg HLM)	125	(Udomnilobol et al., 2023)
K_m (µM)	3	(Udomnilobol et al., 2023)
CES2 V_{max} (pmol/min/mg HIM)	2,347	(Udomnilobol et al., 2023)
K_m (µM)	1.2	(Udomnilobol et al., 2023)
Pathway 3 (DABE to BIBR1087)		
CES1 V_{max} (pmol/min/mg HLM)	26,977	(Udomnilobol et al., 2023)
K_m (µM)	9	(Udomnilobol et al., 2023)
CES2 V_{max} (pmol/min/mg HIM)	301	(Udomnilobol et al., 2023)
K_m (µM)	8.4	(Udomnilobol et al., 2023)
Plasma esterase half-life (min)	364	Measured
**Transport**		
Intestinal P-gp J_{max} (pmol/min)	5,747	Optimized

Parameters	DABE	
	Values	Remarks
K_m (μM)	2.6	(Yamazaki et al., 2019)

*Abbreviations are as follows: ADAM, advanced dissolution absorption and metabolism; B:P, blood-to-plasma concentration ratio; CES, carboxyesterase; CSR, critical saturation ratio; CYP, cytochrome P450; DLM: diffusion layer model; $f_{u,p}$, fraction unbound in plasma; HIM, human intestinal microsome; HLM, human liver microsome; ISEF, intersystem extrapolation factor; J_{max}, maximum transport rate; K_m, Michaelis-Menten constant; Log P, octanol-water partition coefficient; MW, molecular weight; $P_{app,caco-2}$, apparent permeability coefficient across Caco-2 cells; P-gp, P-glycoprotein; pKa, acid dissociation constant; PRC, precipitation rate constant; $V_{d,ss}$, steady-state volume of distribution; V_{max}, maximum velocity.

Supplementary Table 3: Input parameters of BIBR0951 and BIBR1087 models.

Parameters	BIBR0951		BIBR1087	
	Values	Remarks	Values	Remarks
**Physicochemical**				
MW (g/mol)	499.60	ChemDraw	599.7	ChemDraw
Log P	2.65	ChemDraw	4.66	ChemDraw
Compound type	Diprotic base		Ampholyte	
pKa1 / pKa2	4.0 / 12.4	(FDA, 2010)	4.1 / 6.7	(FDA, 2010)
B:P	0.62	Measured	0.61	Measured
$f_{u,p}$	0.277	Measured	0.018	Measured
**Absorption**				
Model	ADAM		N.D.	
PSA[†]	137	(PubChem, 2023)	N.D.	
HBD[†]	3	(PubChem, 2023)	N.D.	
BL global scalar	0.1	Optimized	N.D.	
**Distribution**				
Model	Full PBPK (Method 2)		Full PBPK (Method 2)	
$V_{d,ss}$ (L/kg)	0.31	Predicted	0.12	Predicted
**Elimination**				
Model	Enzyme kinetic		Enzyme kinetic	
Pathway 1 (oxidative metabolism)				
CYP3A4 V_{max} (pmol/min/pmol CYP)	8.3	(Udomnilobol et al., 2023)	N.D.	
K_m (µM)	2.8	(Udomnilobol et al., 2023)	N.D.	
ISEF	0.48	Calculated	N.D.	
CYP3A5 V_{max} (pmol/min/pmol CYP)	1.0	(Udomnilobol et al., 2023)	N.D.	
K_m (µM)	0.6	(Udomnilobol et al., 2023)	N.D.	
ISEF	0.48	Calculated	N.D.	
Pathway 2 (DAB formation)				
CES1 V_{max} (pmol/min/mg HLM)	18,438	(Udomnilobol et al., 2023)	1,050	(Udomnilobol et al., 2023)

Parameters		BIBR0951		BIBR1087	
		Values	Remarks	Values	Remarks
	K_m (μM)	518	(Udomnilobol et al., 2023)	62	(Udomnilobol et al., 2023)
	$f_{u,inc}$	0.1	Adjusted	N.D.	
CES2	V_{max} (pmol/min/mg HIM)	N.D.		7,673	(Udomnilobol et al., 2023)
	K_m (μM)	N.D.		636	(Udomnilobol et al., 2023)
Plasma esterase half-life (min)		55	Measured	N.D.	
$CL_{int,bile}$ (μL/min/million cells)		237	Estimated	N.D.	

*Abbreviations are as follows: ADAM, advanced dissolution absorption and metabolism; BL, basolateral; B:P, blood-to-plasma concentration ratio; CES, carboxyesterase; $CL_{int,bile,}$, intrinsic clearance for biliary excretion; CYP, cytochrome P450; $f_{u,inc}$, fraction unbound in the *in vitro* incubation; $f_{u,p}$, fraction unbound in plasma; HBD, hydrogen bond donor; HIM, human intestinal microsome; HLM, human liver microsome; ISEF, intersystem extrapolation factor; K_m, Michaelis-Menten constant; Log P, octanol-water partition coefficient; MW, molecular weight; pKa, acid dissociation constant; PSA, polar surface area; $V_{d,ss}$, steady-state volume of distribution; V_{max}, maximum velocity; N.D., not determined.

[†] The physicochemical parameters were used for predicting effective permeability coefficient in human (Peff,man) by software following equation: logPeff,man = 4 - 2.546 - 0.011×polar surface area - 0.278×number of hydrogen bond donors (Winiwarter et al., 1998).

Supplementary Table 4: Input parameters of a default DAB model.

Parameters	SV-Dabigatran Values
**Physicochemical**	
MW (g/mol)	471.5
Log P	0.301
Compound type	Ampholyte
pKa1 / pKa2	4.1 / 12.4
B:P	0.648
$f_{u,p}$	0.715
**Distribution**	
Model	Full PBPK (Method 2)
$V_{d,ss}$ (L/kg)	0.85
K_p scalar	2.5
**Elimination**	
Model	Enzyme kinetic
Additional CL_{int} (μL/min/mg HLM)	1.26
CL_R (L/h)	5.85

*Abbreviations are as follows: B:P, blood-to-plasma concentration ratio; CL_{int}, intrinsic clearance; CL_R, renal clearance; $f_{u,p}$, fraction unbound in plasma; HLM, human liver microsome; K_p, tissue-to-plasma partition coefficient; Log P, octanol-water partition coefficient; MW, molecular weight; pKa, acid dissociation constant; $V_{d,ss}$, steady-state volume of distribution.

Supplementary Table 5: Input parameters of perpetrators used in PBPK modeling

Parameters	SV-Clarithromycin (CTC)	Itraconazole (ITZ)*	Hydroxy-itraconazole (OH-ITZ)*	SV-Rifampicin-SD (RF)	SV-Verapamil (VP)	SV-Norverapamil (NVP)
**Physicochemical**						
MW (g/mol)	748	705.6	721.7	823	454.6	440.6
Log P	1.7	4.91	4.1	4.01	4.46	4.66
Compound type	Monoprotic base	Monoprotic base	Monoprotic base	Ampholyte	Monoprotic base	Monoprotic base
pKa1 / pKa2	8.99	3.64	4	1.7 / 7.9	8.78	10.29
B:P	1.0	0.6	0.55	0.9	0.709	0.675
$f_{u,p}$	0.18	0.0015	0.012	0.116	0.090	0.083
**Absorption**						
Model	1st order	1st order	N.D.	ADAM	ADAM	N.D.
$P_{eff,man}$ (10^{-4} cm/sec)	2.12	3.75	N.D.	2.15	6.08	N.D.
**Distribution**						
Model	Minimal PBPK	Minimal PBPK	Full PBPK (Method 2)	Full PBPK (Method 2)	Full PBPK (Method 2)	Minimal PBPK
$V_{d,ss}$ (L/kg)	1.75	4.74	4.72	0.42	5.37	4.139
V_{sac} (L/kg)	N.D.	3.0	2.5	N.D.	N.D.	2
K_p scalar	1	1	1	0.098	1	1
**Elimination**						
Model	Enzyme kinetic	Enzyme kinetic	Enzyme kinetic	Enzyme kinetic	Enzyme kinetic	Enzyme kinetic
Pathways	CYP3A4	CYP3A4	CYP3A4	N.D.	CYP2C8, CYP3A4, CYP3A5	CYP2C8, CYP3A4, CYP3A5
Active hepatic scalar	1	3.5	1	1	1	1
Additional CL (µL/min/mg HLM)	N.D.	N.D.	N.D.	2.8	79.6	N.D.

Parameters	SV-Clarithromycin (CTC)	Itraconazole (ITZ)*	Hydroxy-itraconazole (OH-ITZ)*	SV-Rifampicin-SD (RF)	SV-Verapamil (VP)	SV-Norverapamil (NVP)
$CL_{int,bile}$ (μL/min/million cells)	N.D.	N.D.	N.D.	0.29	N.D.	N.D.
CL_R (L/h)	8.05	N.D.	N.D.	1.26	2.52	1.91
**Transport**						
Intestine	N.D.	N.D.	N.D.	N.D.	P-gp, MRP2	N.D.
Liver	N.D.	N.D.	N.D.	OATP1B1	N.D.	N.D.
**Interaction**						
CYP3A4 $K_{i,u}$ (μM)	8.7	0.001	0.0082	15	N.D.	N.D.
CYP3A5 $K_{i,u}$ (μM)	N.D.	N.D.	N.D.	N.D.	N.D.	N.D.
CYP3A4 $K_{app,u}$ (μM)	12	N.D.	N.D.	N.D.	2.2	10.3
CYP3A4 k_{inact} (1/h)	2.1	N.D.	N.D.	N.D.	2	18
CYP3A5 $K_{app,u}$ (μM)	N.D.	N.D.	N.D.	N.D.	4	4.5
CYP3A5 k_{inact} (1/h)	N.D.	N.D.	N.D.	N.D.	1.8	4.2
P-gp $K_{i,u}$ (μM)	4	2	5	4.3	0.16	0.04

Abbreviations are as follows: ADAM, advanced dissolution absorption and metabolism; B:P, blood-to-plasma concentration ratio; $CL_{int,bile,}$, intrinsic clearance for biliary excretion; CL_R, renal clearance; CYP, cytochrome P450; $f_{u,p}$, fraction unbound in plasma; HLM, human liver microsomes; $K_{app,u}$, unbound concentration for mechanism-based inactivation; $K_{i,u}$, unbound inhibitory constant; k_{inact}, inactivation rate of enzyme; Log P, octanol-water partition coefficient; MRP2, multidrug resistance protein 2; MW, molecular weight; OATP1B1, organic anion transporting polypeptide 1B1; $P_{eff,man}$, effective permeability coefficient in human; P-gp, P-glycoprotein; pKa, acid dissociation constant; $V_{d,ss}$, steady-state volume of distribution; V_{sac}, volume of single adjusting compartment; N.D., not determined.

Supplementary Table 6: Prediction of PK and DDI of plasma DAB following administration of DABE with or without CTC by using the joint PBPK model after step 1 of model development.

Set	DABE		Perpetrators	C_{max} (ng/mL)			$AUC_{0\text{-}inf}$ (ng·h/mL)			References
	Dose	Formulation		Observed	Simulated	Criteria*	Observed	Simulated	Criteria*	
**Training sets**										
1	375 µg	Solution	N.D.	0.17 (0.13-0.23)	0.34	0.09-0.34	1.44 (1.03-2.02)	2.85	0.72-.288	(Prueksaritanont et al., 2017)
1	375 µg	Solution	CTC 500 mg PO BID 5 days	0.79 (0.39-1.61)	1.87	0.40-1.58	5.79 (3.39-9.90)	15.19	2.90-13.38	(Prueksaritanont et al., 2017)
1	DDI magnitudes			4.57 (2.85-7.34)	5.50	2.57-8.14	4.02 (2.99-5.41)	5.32	2.30-7.04	(Prueksaritanont et al., 2017)
2	300 mg	Solution	N.D.	174# (92-310)	795 (315-2,505)	87-348	1,220# (586-2,227)	6,610 (2,399-15,522)	610-2,440	(Delavenne et al., 2013)
2	300 mg	Solution	CTC 500 mg PO BID 5 days	279# (586-2,227)	1,620 (805-3,466)	140-558	1,820# (521-5,000)	13,203 (6,002-25,238)	910-3,640	(Delavenne et al., 2013)
2	DDI magnitudes			1.60#	1.89	1.16-2.20	1.49#	1.94	1.12-1.98	(Delavenne et al., 2013)

*Acceptance ranges for prediction of PK parameters and DDI magnitudes were defined based on the criteria (Guest et al., 2011; Abduljalil et al., 2014). Data were reported as geometric mean (90% confidence interval or % coefficient of variation), except #median (min-max). Notably, the input parameters related to solubility, precipitation, and formulation of DABE were not considered at this step. N.D., not determined.

Supplementary Table 7: Prediction of PK and DDI of plasma DAB following administration of DABE with or without CTC by using the final models.

Set	DABE		Perpetrators	C_{max} (ng/mL)			AUC_{0-inf} (ng·h/mL)			References
	Dose	Formulation		Observed	Simulated	Criteria*	Observed	Simulated	Criteria*	
**Training sets**										
1	375 µg	Solution w/ precipitation	N.D.	0.17 (0.13-0.23)	0.15 (0.14-0.17)	0.09-0.34	1.44 (1.03-2.02)	1.44 (1.30-1.60)	0.72-.288	(Prueksaritanont et al., 2017)
			CTC 500 mg PO BID 5 days	0.79 (0.39-1.61)	0.68 (0.62-0.74)	0.40-1.58	5.79 (3.39-9.90)	6.69 (6.12-7.31)	2.90-13.38	
	DDI magnitudes			4.57 (2.85-7.34)	4.46 (4.17-4.78)	2.57-8.14	4.02 (2.99-5.41)	4.64 (4.33-4.97)	2.30-7.04	
2	300 mg	Solid IR	N.D.	174# (92-310)	166# (40-618)	87-348	1,220# (586-2,227)	1,181# (280-4,538)	610-2,440	(Delavenne et al., 2013)
			CTC 500 mg PO BID 5 days	279# (586-2,227)	295# (75-948)	140-558	1,820# (521-5,000)	2,636# (603-8,734)	910-3,640	
	DDI magnitudes			1.60#	1.61#	1.16-2.20	1.49#	1.97#	1.12-1.98	

*Acceptance ranges for prediction of PK parameters and DDI magnitudes were defined based on the criteria (Guest et al., 2011; Abduljalil et al., 2014). Data were reported as geometric mean (90% confidence interval or % coefficient of variation), except #median (min-max). N.D., not determined.

3 Supplementary Figures

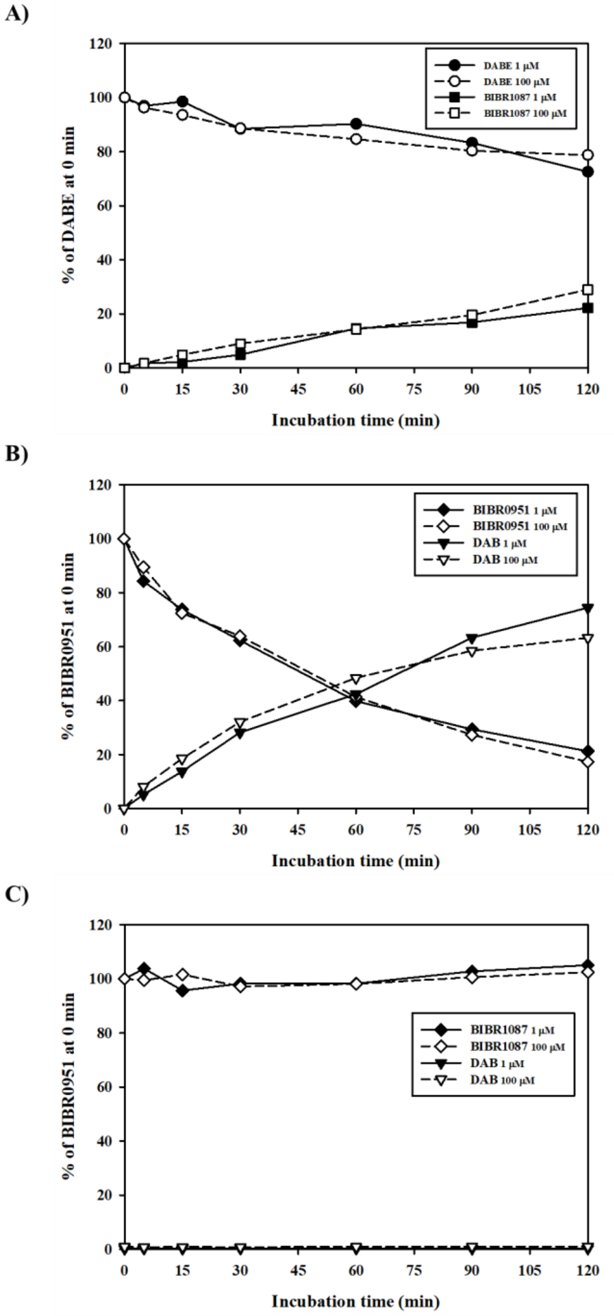

Supplementary Figure 1: Stability of DABE (A), BIBR0951 (B), and BIBR1087 (C) in human plasma. The compounds were represented by the symbols as following: DABE (circle), BIBR0951 (diamond), BIBR1087 (square), DAB (triangle). Black symbols and solid lines represented the incubations with compounds at 1 µM, whereas white symbols and dashed lines represented the incubations with compounds at 100 µM. Data are expressed as % of compounds presented in the incubations, compared to their parents at 0 min (mean, duplicates).

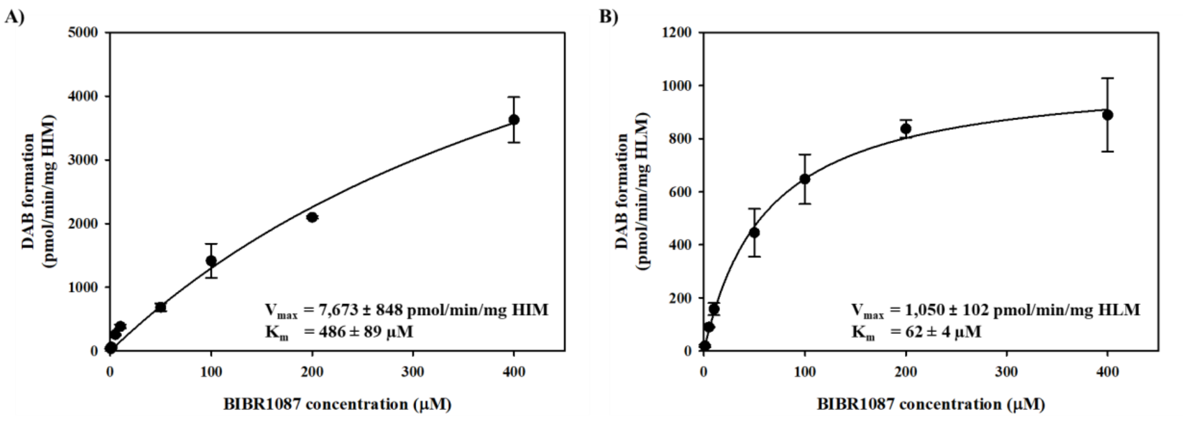

Supplementary Figure 2: Michaelis-Menten kinetic of CES-mediated hydrolysis of BIBR1087 in HIM (A) and HLM (B). Data are expressed as mean ± SD from n=3.

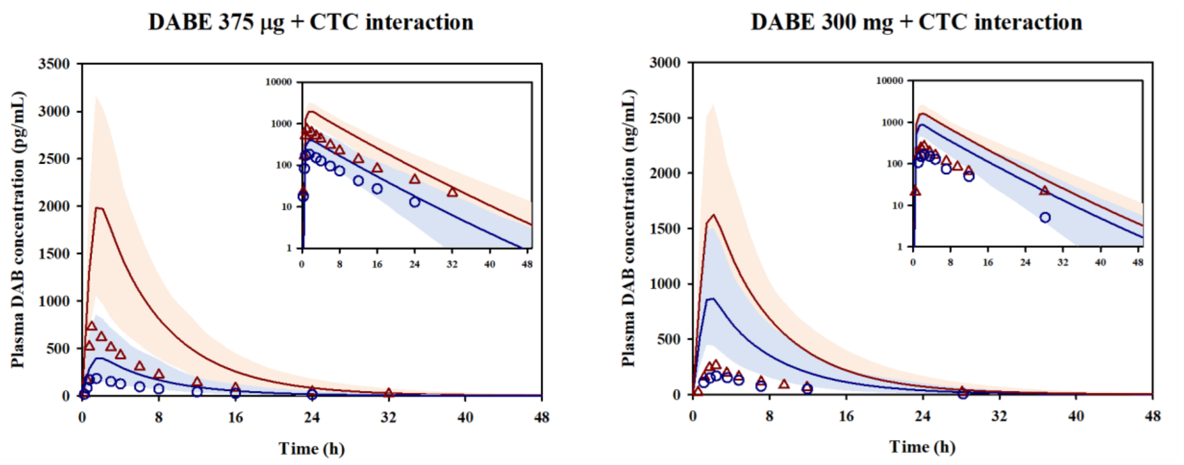

Supplementary Figure 3: Simulated and observed mean plasma concentration-time profiles of DAB following DABE-CTC DDI evaluated at the microdose (375 µg, left panel) or therapeutic dose (300 mg, right panel) of DABE (Delavenne et al., 2013; Prueksaritanont et al., 2017). Graphs depict the simulated DAB profiles after step 1 of model development. The blue circles and lines represent the observed and simulated DAB level following DABE alone, respectively. The red triangles and lines represent the observed and simulated DAB level following DABE-CTC coadministration, respectively. The shaded blue and red areas are the 95% confidence interval of the simulated DAB concentrations in the absence and presence of CTC, respectively.

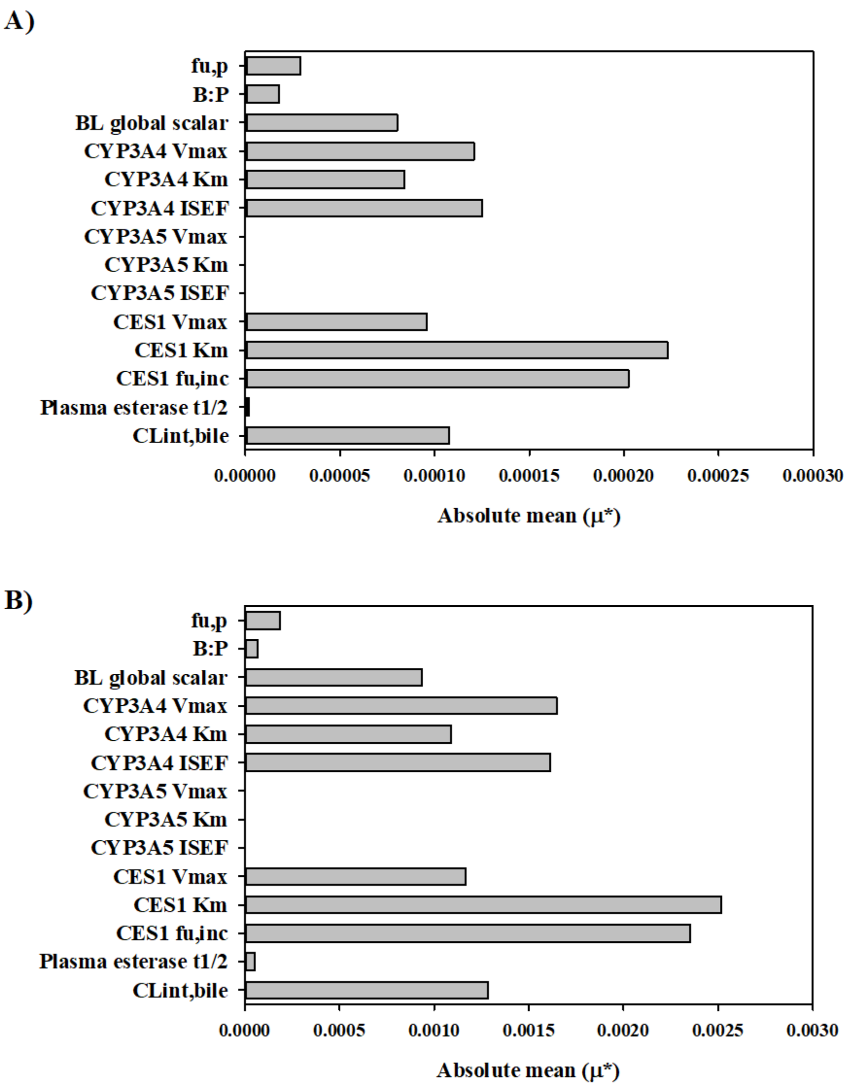

Supplementary Figure 4: Influence of input parameters in the BIBR0951 model on C_{max} (A) and $AUC_{0\text{-}inf}$ (B) of DAB, assessed by a global sensitivity analysis using Morris method. The higher absolute mean (μ*) represents the higher influence of that parameter on the overall simulation outputs.

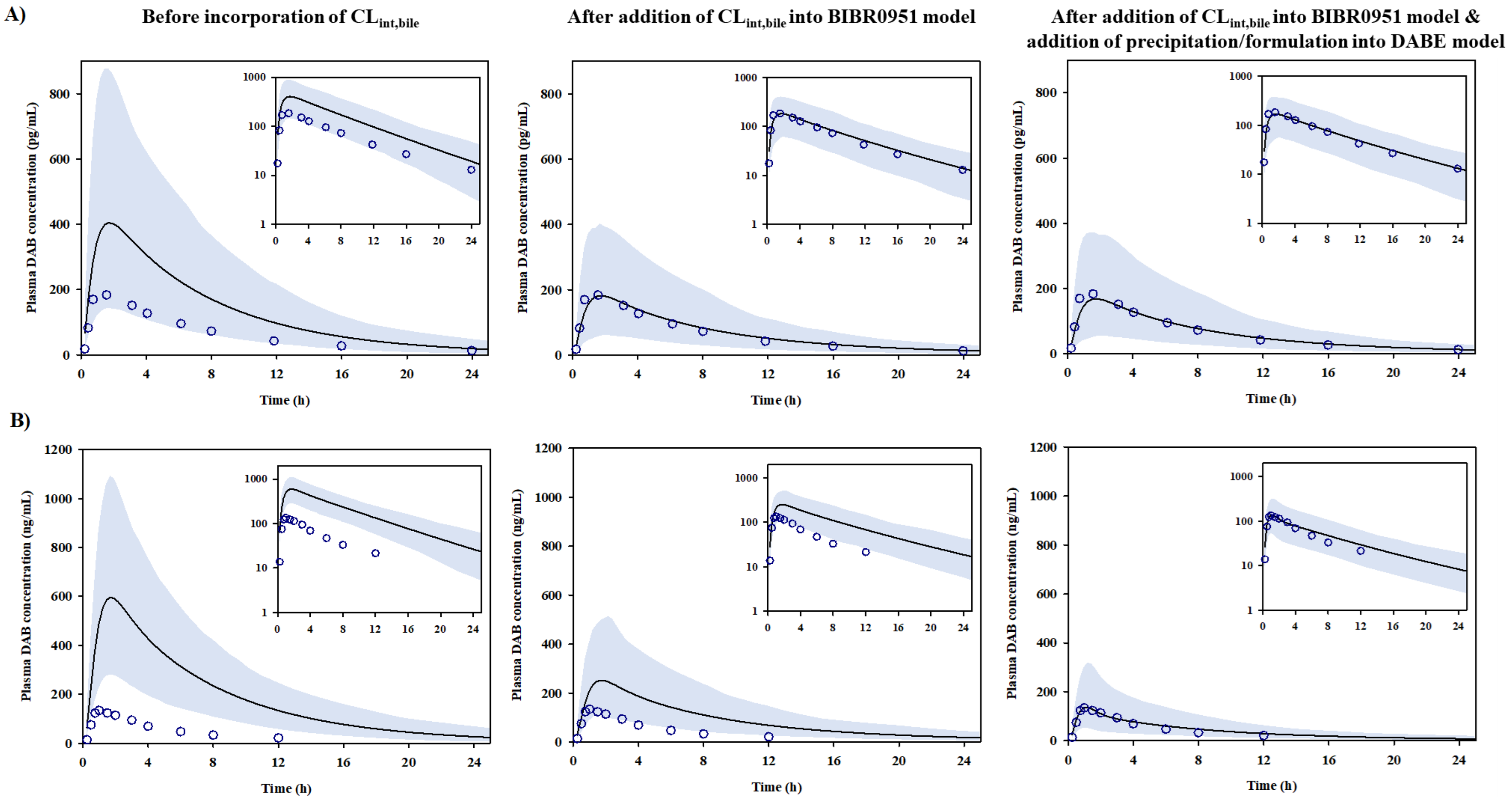

Supplementary Figure 5: Simulated and observed mean plasma concentration-time profiles of DAB following oral DABE administration at the microdose (375 µg; A) and therapeutic dose (300 mg; B) from Prueksaritanont et al. (2017) and Delavenne et al. (2013), respectively. Graphs depict the simulated DAB profiles at different stages of model development: before incorporation of $CL_{int,\ bile}$ (left panel); after addition of $CL_{int,bile}$ into BIBR0951 model (middle panel); after addition of both $CL_{int,bile}$ and precipitation/formulation into BIBR0951 and DABE models, respectively (right panel). The black lines and blue circles represent the simulated and observed DAB levels, respectively. The shaded blue areas are the 95% confidence interval of the simulated results.

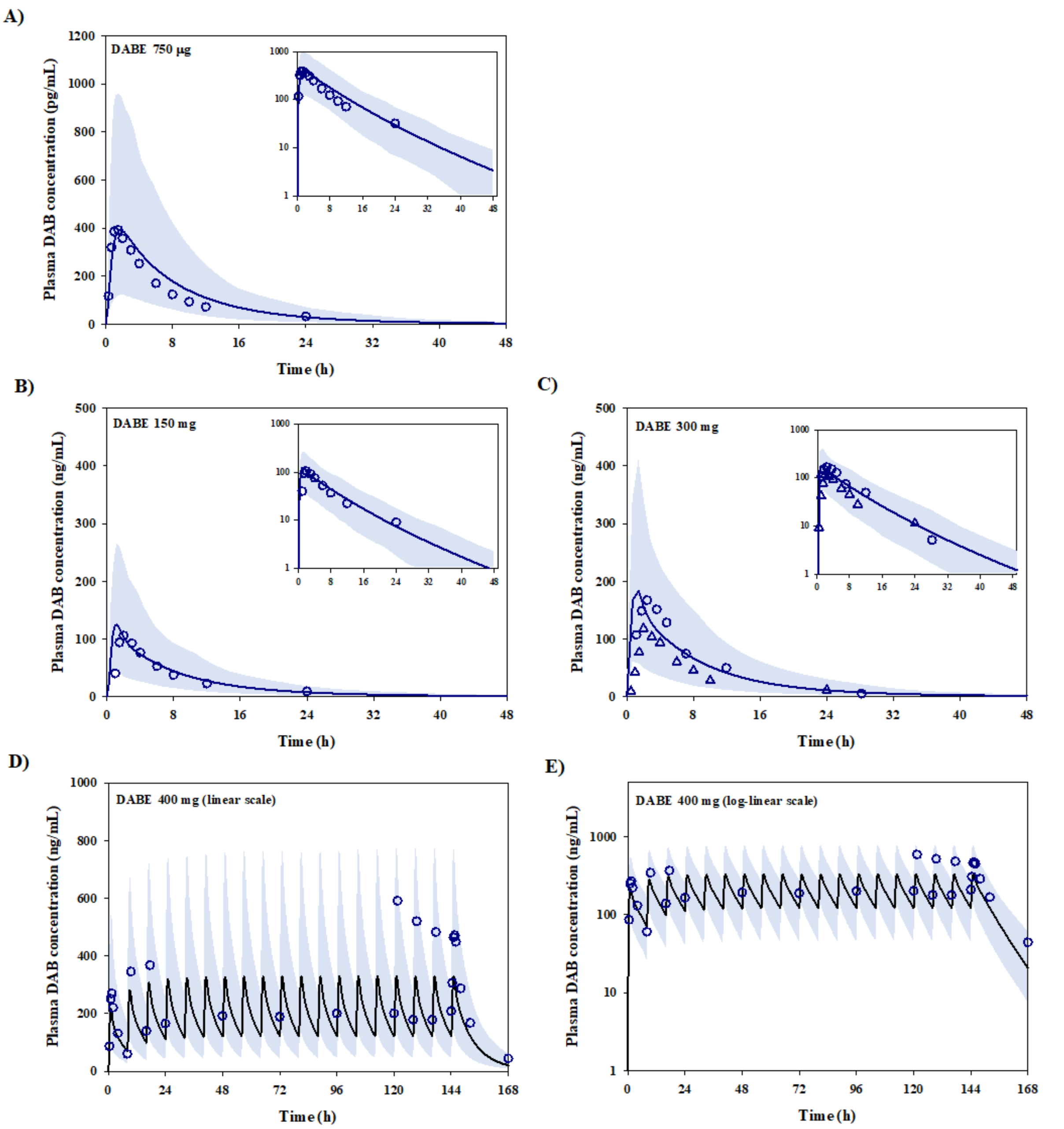

Supplementary Figure 6: Simulated and observed mean plasma concentration-time profiles of DAB following single and multiple oral administration of DABE at 750 µg (A), 150 mg (B), 300 mg (C), and 400 mg (D and E) from Rattanacheeworn et al. (2021), Stangier et al. (2008), Gouin-Thibault et al. (2017), and Stangier (2008), respectively. The blue circles and lines represent the observed and simulated DAB level, respectively. The shaded blue areas are the 95% confidence interval of the simulated DAB concentrations.

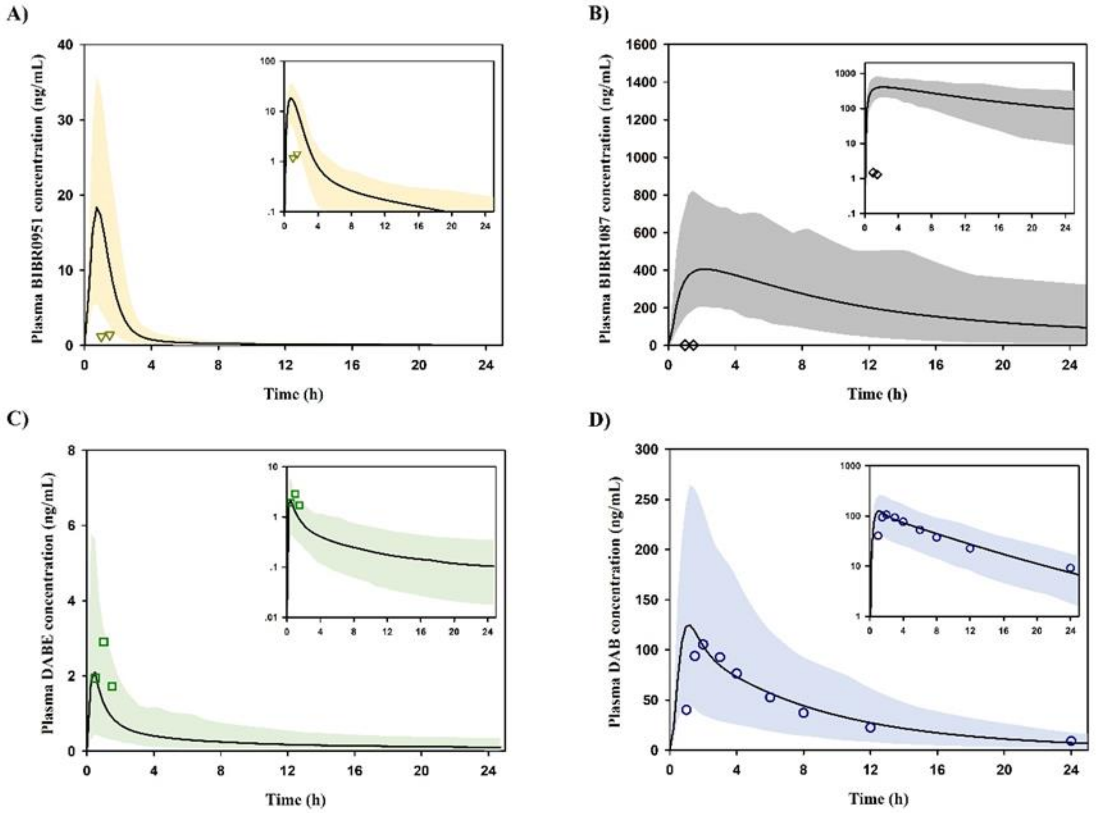

Supplementary Figure 7: Simulated and observed mean plasma concentration-time profiles of BIBR0951 (A), BIBR1087 (B), DABE (C), and DAB (D) following oral administration of DABE 150 mg capsule from Stangier et al. (2008). Graphs are expressed as the linear (main graph) and semilogarithmic (inset) scales. The lines and symbols represent simulated and observed data, respectively. The shaded grey areas are the 95% confidence interval of the simulated data. Notably, the observed data of DAB were from plasma total DAB (free + conjugated forms).

4 References for supplementary information

Abduljalil K, Cain T, Humphries H, and Rostami-Hodjegan A (2014) Deciding on success criteria for predictability of pharmacokinetic parameters from in vitro studies: an analysis based on in vivo observations. *Drug Metab Dispos* **42:**1478-1484.

Blech S, Ebner T, Ludwig-Schwellinger E, Stangier J, and Roth W (2008) The metabolism and disposition of the oral direct thrombin inhibitor, dabigatran, in humans. *Drug Metab Dispos* **36:**386-399.

Delavenne X, Ollier E, Basset T, Bertoletti L, Accassat S, Garcin A, Laporte S, Zufferey P, and Mismetti P (2013) A semi-mechanistic absorption model to evaluate drug-drug interaction with dabigatran: application with clarithromycin. *Br J Clin Pharmacol* **76:**107-113.

Doki K, Neuhoff S, Rostami-Hodjegan A, and Homma M (2019) Assessing potential drug-drug interactions between dabigatran etexilate and a p-glycoprotein inhibitor in renal impairment populations using physiologically based pharmacokinetic modeling. *CPT Pharmacometrics Syst Pharmacol* **8:**118-126.

Farhan N, Cristofoletti R, Basu S, Kim S, Lingineni K, Jiang S, Brown JD, Fang LL, Lesko LJ, and Schmidt S (2021) Physiologically-based pharmacokinetics modeling to investigate formulation factors influencing the generic substitution of dabigatran etexilate. *CPT Pharmacometrics Syst Pharmacol* **10:**199-210.

FDA (2010) US Food and Drug Administration. Pradaxa: Chemistry review(s). *Center for Drug Evaluation and Research (CDER).*

Gouin-Thibault I, Delavenne X, Blanchard A, Siguret V, Salem JE, Narjoz C, Gaussem P, Beaune P, Funck-Brentano C, Azizi M, Mismetti P, and Loriot MA (2017) Interindividual variability in dabigatran and rivaroxaban exposure: contribution of

ABCB1 genetic polymorphisms and interaction with clarithromycin. *J Thromb Haemost* **15:**273-283.

Guest EJ, Aarons L, Houston JB, Rostami-Hodjegan A, and Galetin A (2011) Critique of the two-fold measure of prediction success for ratios: application for the assessment of drug-drug interactions. *Drug Metab Dispos* **39:**170-173.

Hartter S, Sennewald R, Nehmiz G, and Reilly P (2013) Oral bioavailability of dabigatran etexilate (Pradaxa®) after co-medication with verapamil in healthy subjects. *Br J Clin Pharmacol* **75:**1053-1062.

Imai T, Isozaki M, and Ohura K (2022) Esterases Involved in the Rapid Bioconversion of Esmolol after Intravenous Injection in Humans. *Biol Pharm Bull* **45**:1544–1552.

Ishiguro N, Kishimoto W, Volz A, Ludwig-Schwellinger E, Ebner T, and Schaefer O (2014) Impact of endogenous esterase activity on in vitro p-glycoprotein profiling of dabigatran etexilate in Caco-2 monolayers. *Drug Metab Dispos* **42:**250-256.

Proctor NJ, Tucker GT, and Rostami-Hodjegan A (2004) Predicting drug clearance from recombinantly expressed CYPs: intersystem extrapolation factors. *Xenobiotica* **34**:2, 151-178.

Prueksaritanont T, Tatosian DA, Chu X, Railkar R, Evers R, Chavez-Eng C, Lutz R, Zeng W, Yabut J, Chan GH, Cai X, Latham AH, Hehman J, Stypinski D, Brejda J, Zhou C, Thornton B, Bateman KP, Fraser I, and Stoch SA (2017) Validation of a microdose probe drug cocktail for clinical drug interaction assessments for drug transporters and CYP3A. *Clin Pharmacol Ther* **101:**519-530.

PubChem (2023) Compound Summary for CID 446804, Dabigatran ethyl ester. *National Center for Biotechnology Information*

Rattanacheeworn P, Kerr SJ, Kittanamongkolchai W, Townamchai N, Udomkarnjananun S, Praditpornsilpa K, Thanusuwannasak T, Udomnilobol U, Jianmongkol S,

Ongpipattanakul B, Prueksaritanont T, Avihingsanon Y, and Chariyavilaskul P (2021) Quantification of CYP3A and drug rransporters activity in healthy young, healthy elderly and chronic kidney disease elderly patients by a microdose cocktail approach. *Front Pharmacol* **12:**726669.

Stangier J (2008) Clinical pharmacokinetics and pharmacodynamics of the oral direct thrombin inhibitor dabigatran etexilate. *Clin Pharmacokinet* **47:**285-295.

Stangier J, Stahle H, Rathgen K, Roth W, and Shakeri-Nejad K (2008) Pharmacokinetics and pharmacodynamics of dabigatran etexilate, an oral direct thrombin inhibitor, are not affected by moderate hepatic impairment. *J Clin Pharmacol* **48:**1411-1419.

Udomnilobol U, Jianmongkol S, and Prueksaritanont T (2023) The potentially significant role of CYP3A-mediated oxidative metabolism of dabigatran etexilate and its intermediate metabolites in drug-drug interaction assessments using microdose dabigatran etexilate. *Drug Metab Dispos* **51**:1216-1226.

Winiwarter S, Bonham NM, Ax F, Hallberg A, Lennernäs H, and Karlén A (1998) Correlation of Human Jejunal Permeability (in Vivo) of Drugs with Experimentally and Theoretically Derived Parameters. A Multivariate Data Analysis Approach. *J. Med. Chem.* **41**:4939-4949.

Yamazaki S, Costales C, Lazzaro S, Eatemadpour S, Kimoto E, and Varma MV (2019) Physiologically-based pharmacokinetic modeling approach to predict rifampin-mediated intestinal p-glycoprotein induction. *CPT Pharmacometrics Syst Pharmacol* **8:**634-642.

Yu S, Li S, Yang H, Lee F, Wu JT, and Qian MG (2005) A novel liquid chromatography/tandem mass spectrometry based depletion method for measuring red blood cell partitioning of pharmaceutical compounds in drug discovery. *Rapid Commun Mass Spectrom* **19:**250-254.
